# The impact of socioeconomic factors on the incidence and characteristics of first-episode psychosis

**DOI:** 10.1017/S2045796025100206

**Published:** 2025-09-02

**Authors:** Martino Belvederi Murri, Alice Onofrio, Chiara Punzi, Nicola Caranci, Enrico Rubolino, Francesco Giovinazzi, Danila Azzolina, Federica Folesani, Luigi Grassi, Ilaria Tarricone, Fabrizio Starace

**Affiliations:** 1 Department of Neuroscience and Rehabilitation, Institute of Psychiatry, University of Ferrara, Ferrara, Italy; 2 Department of Innovation in Health and Social Care, General Directorate of Health and Welfare, Emilia-Romagna Region, Bologna, Italy; 3 Department of Business and Economics, University of Lausanne, Lausanne, France; 4 Department of Mental Health and Pathological Addiction, AUSL Modena, Modena, Italy; 5 Department of Environmental and Preventive Science, University of Ferrara, Ferrara, Italy; 6 Department of Medical and Surgical Sciences, Bologna Transcultural Psychosomatic Team (BoTPT), University of Bologna, Bologna, Italy

**Keywords:** epidemiology, minority issues and cross cultural psychiatry, psychoactive substance use disorder, psychosis, social factors

## Abstract

**Aim:**

The environment shapes the risk of psychosis. In particular, urbanicity, deprivation or inequality, migrant density and cannabis availability may not only influence psychosis incidence, but also the characteristics of individuals who arrive at clinical services. This study examined how socioeconomic factors influence the incidence and characteristics of cases of First-Episode Psychosis (FEP).

**Methods:**

We analysed prospective data collected from the FEP early detection programme of Emilia-Romagna, a high-income Italian region. Participants were 1240 individuals aged 18–35 years, who presented at the public healthcare services for a FEP. Exposures were derived from area-level data of 331 municipalities. We used population density, socioeconomic deprivation, educational deprivation, economic inequality, migrant density (proportion of migrants), frequent cannabis use (proportion of people aged 15–19 years old who reported frequent cannabis use). Outcome measures were FEP incidence (cases/100 000 inhabitants at risk per year) and characteristics (age of onset, migrant status, unemployment, substance use, treatment lag [DUP], family and resource problems). We reviewed pertinent literature, and formulated a Directed Acyclic Graph to present causal assumptions and provide adjustment sets for Bayesian spatial and multilevel models of social causation. To compare the effects of different exposures, we computed Average Marginal Effects and report the outcome changes that correspond to one standard deviation change of the exposure, incidence rate ratios (IRR) or odds ratios (OR).

**Results:**

The exposures and incidence of FEP displayed heterogeneous spatial distribution, with no spatially organized pattern. Accordingly, incidence and characteristics were best modelled as non-spatial, three-level hierarchical models. The incidence of FEP was influenced by population density (IRR, 1.14; 95% CrI, 1.03; 1.29), educational deprivation (IRR, 1.15; 95% CrI, 1.02; 1.31) and frequent cannabis use (IRR, 1.31; 95% CrI, 0.98; 1.82), more than socioeconomic deprivation. Higher migrant density in an area shortened the DUP on average by 3.4 months (95% CrI, −1.122; 0.76), while an increase of cannabis use of one standard deviation increased the DUP of 12.9 months (95% CrI, −2.86; 6229). Socioeconomic deprivation increased the likelihood of FEP cases being substance users (OR, 1.12; 95% CrI, 1.01; 1.26), while population density decreased it (OR, 0.91; 95% CrI, 0.83; 1.00).

**Conclusions:**

Area-level socioeconomic features affect both the incidence and the characteristics of FEP, including the probability of individual being migrants, substance users or having a different DUP. Educational deprivation may function as a proxy for culture- or cognitive-related factors. Area-level socioeconomic data may inform public healthcare strategies for early identification and availability of tertiary clinical services.

## Introduction

The socioeconomic environment influences the incidence of psychosis, and the clinical profiles of affected individuals. Nonetheless, research on this topic is still warranted, given its clinical and societal importance.

Psychotic disorders have a multifaceted aetiology, at the intersection between genetic predisposition and environmental factors. Particular attention is needed to how social, economic and cultural factors shape their impact across the globe (D’Andrea *et al*., 2024; Jongsma *et al*., 2018; Kirkbride *et al*., 2024). Research began to delineate how specific socioeconomic factors may influence the presentation of psychotic disorders: some trends have emerged in relation to the urban vs. rural settings (Kirkbride *et al*., 2017), population ethnic composition (Chung *et al*., 2023), absolute societal deprivation as well as relative economic inequalities (Kirkbride *et al*., 2014; Logeswaran *et al*., 2023).

Demonstrating these effects, however, is challenging because of inherent geographic differences, which imply methodological constraints and complex causal interpretation (Grover *et al*., 2024). For instance, the link between urbanicity and increased incidence of First-Episode Psychosis (FEP) is not consistent worldwide, and exhibits large geographic differences (Jongsma *et al*., 2018) partly related to the level of analysis (neighbourhood or larger levels), partly because of distinct historical and sociocultural features of each area, that delineate unique ‘etiological’ landscapes (DeVylder *et al*., 2018; Kirkbride *et al*., 2018). In addition, research needs to address the complex causal pathways that underlie social causation, the reciprocal interplay between factors, both at the area-level and individual level, and their distributions and non-linear patterns (González-Valderrama *et al*., 2022). Notably, recent studies robustly support social causation pathways of psychosis, even accounting for the role of social immobility, social constraints (Logeswaran *et al*., 2023), and genetic factors (Maxwell *et al*., 2021). The synthesis of data across distinct domains proves particularly effective to predict the onset of psychosis within communities: a recent study harnessed a range of social, economic, and demographic factors—including age, gender, ethnicity, small-area-level deprivation, social fragmentation, and regional cannabis use—and accurately predicted the incidence of FEP cases across a broad UK region (McDonald *et al*., 2021). A similar approach could also be applied to explore the impact of socioeconomic factors on the characteristics of FEP users, offering valuable insights for resource allocation and the customization of care.

### Aims

The aim of this study was to examine the effects of socioeconomic factors on the treated incidence of first-episode psychosis, and user characteristics. We hypothesized that area-level socioeconomic indices would predict not only the variability of treated incidence, but also key features of presenting users, such as, age of onset, migrant status, unemployment, substance use, treatment lag, family or resource problems.

## Methods

### Study design and setting

This study uses data of FEP cases collected for the Regional Program for the detection and care of FEP (RER-FEP) between 1 January 2013 and 30 December 2019, first 7 years of activity. The programme was developed within the public health system for the early detection of FEP cases and multi-component care (Regione Emilia Anglin *et al*., 2023; Belvederi Murri *et al*., 2021; Romagna, 2016). Data were linked with those from the regional administrative database of the Emilia-Romagna Regional Mental Healthcare Office, in the same timeframe, using a unique anonymized regional identifier assigned to each user across health records.

Emilia-Romagna is a region in northern Italy with 4.5 million inhabitants and one of the highest GDP per capita in the country, ranking among the most affluent regions in Europe. It includes large urban areas such as Bologna, Modena and Parma, and a mix of smaller towns and rural municipalities. The region has a well-developed public health system and a high standard of living. Around 12% of the population is of foreign origin.

### Inclusion criteria

Eligibility was based on the presence of a FEP. Admissible ICD-9 diagnoses were affective psychoses (295.34, 296.24, 296.44, 296.14, 296.54, 296.64) and non-affective psychosis (295.0–295.95, 299.9, 297, 298), excluding substance-induced psychosis. Diagnoses were checked regardless of whether they were recorded as primary or secondary. Other criteria were age between 18 and 35 years; absence of prior treatment with antipsychotics lifetime, absence of intellectual disability. Data include also individuals who were ineligible for the RER-FEP programme and were referred to the CMHC for treatment as usual. Thus, individuals without Italian language proficiency who were excluded from the program, were included in this study.

Ethical approval was obtained by the Area Vasta Emilia Centro Ethical Committee (CE-AVEC); the study conforms to principles expressed in the Declaration of Helsinki. Participants gave their written informed consent prior to their inclusion. This study followed the Strengthening the Reporting of Observational Studies in Epidemiology (STROBE) reporting guideline.

### Assessments and outcome definitions

Treated incidence was estimated as the number of FEP cases who had access to care per 100 000 persons at risk (aged 18–35 years) per year, per gender, within each municipality. Yearly data of at-risk population were retrieved from the national census (ISTAT). Participants underwent the collection of basic sociodemographic and clinical data, including migrants status, first-generation (born outside Italy) or second-generation (born in Italy from parents born abroad). Presence of substance use (any problematic use of psychoactive substance use) was rated as present by clinicians during clinical contacts, in most cases aided by toxicologic screens. Similarly, clinicians estimated the duration of untreated psychosis (DUP) in months, defined as the time elapsing between the emergence of clear psychotic symptoms and initiation of antipsychotic treatment. The date of onset of psychosis was derived from retrospective clinical interviews. Participants were assessed by psychiatrists using the Italian version of the Health of the Nation Outcome Scales (HoNOS) (Belvederi Murri *et al*., 2021; Lora *et al*., 2001). This study analysed in particular items pertaining *Problems with relationships* (item 9), *Activities of daily living and (or) self-care* (10), *Living conditions and (or) family life* (11) and *Occupation and (or) school attendance* (12) to obtain measures of individual social functioning and resource availability *(Supplementary Methods, paragraph S1.3)*.

### Predictors

We examined the role of population density (log transformed), global deprivation, educational deprivation, economic inequality, migrant density (indexed by proportion of migrants in each municipality, on the total resident population), cannabis use. Data on density and percentage of people born outside Italy over the total population was obtained from the Italian Institute of statistics and those on deprivation from the ISTAT 2011 census. The set of deprivation indices from the social, economic, housing and education domains, at the level of municipality, was obtained from ISTAT data as defined by a recent study (Caranci *et al*., 2010; Rosano *et al*., 2020). We used data of the global deprivation index and an educational deprivation index (percentage of the population aged 15–60 years having elementary education or less). The economic inequality index was computed as the ratio between the subjects in the highest income brackets and subjects in the lowest bracket (Piketty and Saez, 2014). Cannabis use was estimated using 2019 ESPAD survey data on high school students (aged 15–19 years), reporting the provincial-level percentage of individuals using cannabis ≥20 times in the past 30 days (Benedetti *et al*., 2021). Further details on predictors, outcomes and data reduction is reported in the supplement (S1.1–S1.4).

### Data analysis

#### Causal inference

First, we conducted analyses on the effects of socioeconomic variables on incidence, then, on demographic and clinical characteristics of participants. Data analysis was based on a causal inference framework (McElreath, 2018) to support estimation of the effects of interest and identification of variables for model adjustments. We developed a Directed Acyclic Graph (DAG, par. S1.4, Supplementary Fig. 1, SF1) to depict assumptions on causal relationships between socioeconomic factors, psychosis incidence and individual characteristics in the study, supported by a literature review. We then used the DAG to categorize variables as confounders, colliders or mediators for each model. For clarity, we define confounders as variables that causally influence both exposure and outcome, colliders as variables influenced by both, and mediators as variables lying on the causal pathway between exposure and outcome. We adjusted each predictor-outcome analysis for the confounders that were identified by the DAG (Supplementary Table 1, ST1). Unless otherwise specified, we included random slopes for adjustment variables to account for potential variations in their effects across municipalities, in line with recommendations (Barr *et al*., 2013).

**Figure 1.**
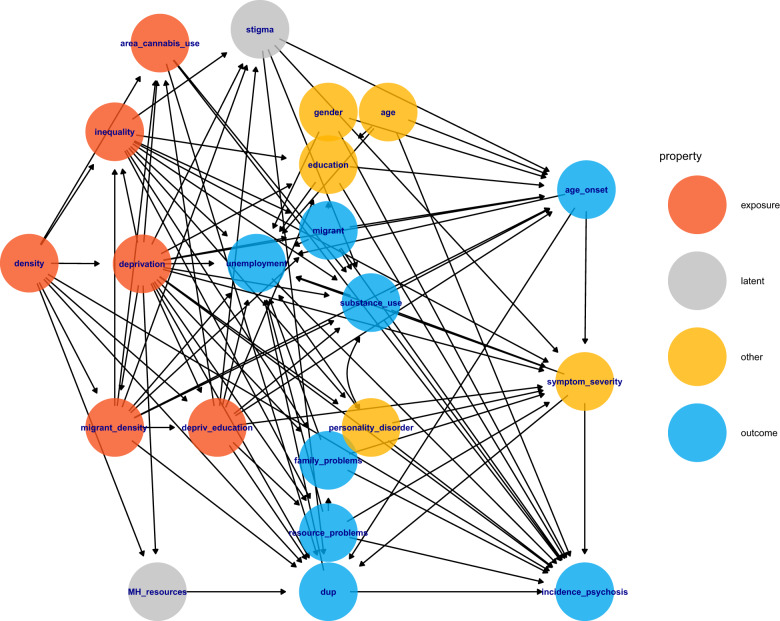
Directed Acyclic Graph representing the causal assumptions of the study.

#### Effect estimation

Data was analysed with Bayesian Generalized Multilevel Models in *brms* (Bürkner, 2017), with outcome-specific distributions: incidence was modelled using a negative binomial distribution to estimate incidence rate ratios (IRRs), since overdispersion was expected (Belvederi Murri *et al*., 2023). Binary outcomes (e.g., substance use, migrant status) were modelled using a Bernoulli distribution with logit link to estimate odds ratios (ORs); DUP was modelled with a lognormal distribution to account for its positive skew. (Supplement, Par. 1.5). The association between each couple of exposure and outcome was analysed in distinct models of increasing complexity, with municipalities nested in provinces, plus a spatial modified Besag York Mollié (BYM2) model, which accounts for spatial autocorrelation and can identify spatial clusters of outcome distribution (Morris *et al*., 2019).

The paper reports the exposure–outcome associations that had high credibility, i.e. where the posterior distribution suggested 95% or higher probability of the effect being non-zero (Probability of Direction, PD). The supplement reports those with lower level of credibility (89–95% PD). We estimated the average marginal effects with their 95% credible intervals (CrI) using *marginal effects* (Arel-Bundock, 2023). In particular, the entity of change of the outcome that would correspond (all else equal) to a change of the exposure spanning: (a) the whole exposure value (minimum to maximum level based on the observed range in the data); (b) one standard deviation around the mean (1SD). Effects are reported both on the response scale and as ratios (IRRs and ORs). Of note, estimates from the observed minimum–maximum range may enhance interpretability, but be more sensitive to outliers.

The *marginal effects* package reports 95% Equal-Tailed credible Intervals (ETIs), which are symmetric around the median and model-agnostic. However, to better capture where the bulk of the posterior probability lies we additionally computed the probability of direction using the *bayestestR* package, which is based on high-density intervals (HDIs). Credibility assessments are based on HDIs (Makowski *et al*., 2019).

## Results

### Causal assumptions and development of the DAG

After the literature review, we built the DAG representing assumptions on the causal relationships between socioeconomic indices and FEP incidence/characteristics (Supplement, Par. S2.1-S2.2). We assumed that population density would drive general deprivation, educational deprivation, inequality and area-level migrant density; such indices would in turn increase the incidence of FEP through increases of unemployment, individual problems, educational attainment, area-level and individual substance use, increased severity of symptoms. In addition, we assumed that socioeconomic disadvantage would also increase the incidence of FEP through a relative lack of resources in the mental health system and increased stigma (Fig. 1). Analysis of the DAG provided the minimal adjustment sets for each model (ST1).

### Characteristics of municipalities in the Emilia-Romagna region

**Figure 2.**
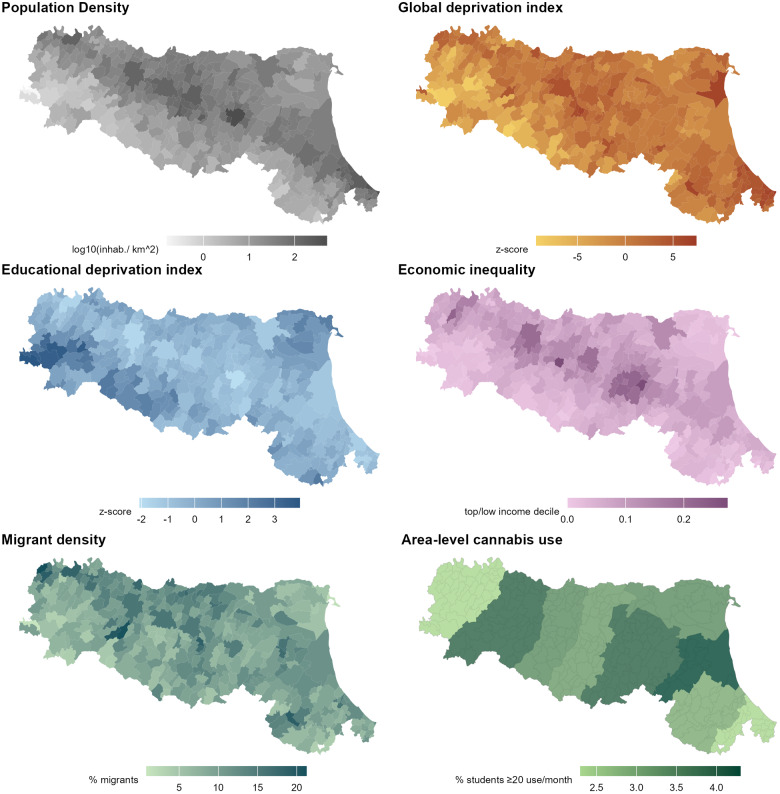
Distribution of socioeconomic factors in the Emilia-Romagna region.

The distribution of socioeconomic indices was highly variable across the 331 municipalities and nine provinces (Table 1 and Fig. 2). As expected, socioeconomic indices were inter-correlated: population density correlated with global deprivation (R = 0.64), inequality (R = 0.54), proportion of migrants (R = 0.42) and was weakly negatively correlated with educational deprivation (R = −0.10, so that higher density municipalities displayed higher education, consistent with the presence of universities and higher concentrations of younger, more educated residents in larger municipalities in this region). The global deprivation index had a weak positive correlation with inequality (R = 0.13), educational deprivation (R = 0.28) and migrant density (0.38, all *P* < 0.05). In turn, educational deprivation was negatively correlated with economic inequality, so that areas with lower education had greater inequality (R = 0.30). Frequent cannabis use only correlated with economic inequality (R = 0.26). The full correlation matrix is reported in SF3, the distribution across provinces in SFs 4–11.

**Table 1.** Characteristics of municipalities grouped by province

Characteristic	Bologna, *N* = 55^1^	Ferrara, *N* = 23^1^	Forlì-cesena, *N* = 30^1^	Modena, *N* = 47^1^	Parma, *N* = 45^1^	Piacenza, *N* = 46^1^	Ravenna, *N* = 18^1^	Reggio emilia, *N* = 42^1^	Rimini, *N* = 25^1^
Area, mean (SD), km^2^	69 (43)	118 (105)	81 (66)	59 (38)	79 (48)	58 (33)	106 (153)	56 (53)	35 (29)
Population at risk, mean (SD), thousands	3.2 (9.9)	2.4 (4.3)	2.3 (4.6)	2.8 (5.3)	1.9 (5.6)	1.1 (2.8)	3.7 (6.2)	2.4 (5.1)	2.5 (5.4)
Population density, Mean (SD), No./km^2^	44 (79)	20 (19)	43 (61)	48 (52)	20 (24)	19 (26)	37 (16)	46 (32)	84 (118)
**Deprivation indices^a^**									
Global deprivation index	0.6 (1.8)	0.0 (2.4)	1.2 (2.6)	0.3 (2.8)	−1.6 (3.3)	−2.2 (3.4)	−1.0 (1.6)	0.5 (2.3)	3.7 (2.3)
Educational deprivation index	−0.48 (0.75)	1.00 (1.34)	−0.18 (0.70)	0.30 (0.90)	0.00 (0.94)	−0.09 (1.11)	−0.30 (0.57)	0.47 (0.91)	−0.62 (0.60)
Unemployment index	−0.09 (0.66)	0.40 (0.87)	−0.08 (1.05)	−0.11 (0.76)	−0.45 (0.68)	−0.41 (1.41)	−0.33 (0.80)	0.29 (0.73)	1.38 (0.88)
House occupation index	0.38 (0.67)	−0.54 (0.55)	0.78 (0.72)	0.13 (0.75)	−0.70 (0.95)	−0.74 (1.02)	−0.44 (0.68)	0.13 (0.63)	1.40 (0.72)
Housing index	0.36 (0.76)	−0.34 (0.60)	0.43 (0.85)	−0.04 (1.03)	−0.09 (1.34)	−0.06 (1.30)	0.04 (0.57)	0.00 (0.75)	−0.44 (0.73)
Single-parent index	0.45 (0.56)	−0.56 (0.58)	0.22 (0.72)	0.03 (0.81)	−0.38 (0.77)	−0.86 (0.75)	0.02 (0.40)	−0.37 (0.56)	1.99 (1.08)
Income 0 to 10 000 euros, mean (SD)^b^	20.3 (2.5)	27.6 (8.3)	26.4 (3.3)	24.2 (3.7)	25.2 (6.2)	27.2 (6.1)	24.3 (2.4)	22.8 (2.9)	32.4 (3.5)
Income > 75 000 euros, mean (SD)^b^	2.01 (0.94)	0.92 (0.60)	1.11 (0.56)	1.65 (0.76)	1.69 (0.89)	1.66 (0.94)	1.48 (0.48)	1.87 (0.76)	1.04 (0.62)
Inequality index, mean (SD)^c^	0.10 (0.06)	0.04 (0.03)	0.04 (0.02)	0.07 (0.04)	0.08 (0.05)	0.07 (0.04)	0.06 (0.02)	0.09 (0.04)	0.03 (0.02)
Migrant density, proportion of migrants over total population, mean % (SD)	9.6 (2.2)	7.7 (2.7)	10.3 (3.3)	11.3 (3.2)	10.6 (3.9)	10.1 (4.5)	11.9 (2.4)	10.4 (3.2)	8.2 (2.5)
Lifetime cannabis use, %^d^	39.00	33.80	35.10	33.80	34.00	32.10	37.80	34.10	35.30
Frequent cannabis use, males and females, %	6.0 (m), 1.7 (f)	4.8 (m), 1.7 (f)	4.3 (m), 1.4 (f)	4.9 (m), 1.1 (f)	5.5 (m), 2.2 (f)	3.7 (m), 0.9 (f)	6.4 (m), 2.0 (f)	5.0 (m),1.4 (f)	3.9 (m),0.6 (f)
Total FEP cases over 7 years, No.	268	101	83	197	220	65	35	197	74
FEP cases/municipality over 7 years, mean (SD)	5 (17)	4 (9)	3 (6)	4 (7)	5 (16)	1 (4)	2 (5)	5 (8)	3 (8)

a *z*-Scores. ^b^Income brackets, percentage of individuals in the bracket, over the total number of taxpayers. ^c^Ratio of the previous income brackets. ^d^Data from the ESPAD survey of cannabis use among 15–19 students in the area.

### Analysis of clinical data

In the study period 1240 cases of FEP met inclusion criteria (70.5% males, Table 2). Considering an average population at risk of 797 thousand individuals per year, the crude average incidence rate of FEP was 21.7 new cases per 100 000 persons/year (95% Confidence Interval [CrI], 19.3–24.1). As expected the incidence rate was higher for males (30.9 cases; 95%CrI: 26.5–35.2) than females (12.5 cases; 95% CrI: 10.4–14.5). The characteristics of participants with FEP displayed a substantial variability across the provinces and municipalities of the region (Table 2).

**Table 2.** Characteristics of participants with FEP

	F, *N* = 366^a^	M, *N* = 874^a^	*P*-value^b^
Age			0.049
Mean (SD)	24.4 (5.2)	23.6 (4.6)	
Education			0.005
Elementary/None	8 (2.6%)	20 (2.7%)	
Lower secondary	101 (33%)	293 (40%)	
Upper secondary	155 (50%)	362 (50%)	
Graduate	44 (14%)	55 (7.5%)	
Unknown	58	144	
Civil status			<0.001
Single	265 (85%)	716 (96%)	
Married/Cohabiting	41 (13%)	27 (3.6%)	
Separated/Divorced	4 (1.3%)	2 (0.3%)	
Unknown	56	129	
1st or 2nd generation migrant	90 (25%)	209 (24%)	0.8
Occupation			<0.001
Other	156 (50%)	263 (35%)	
Unemployed	109 (35%)	326 (44%)	
Non-working	2 (0.6%)	3 (0.4%)	
Employed	47 (15%)	156 (21%)	
Unknown	52	126	
Living arrangement			0.3
Other co-living	34 (11%)	83 (11%)	
Other	3 (1.0%)	9 (1.2%)	
Acquired family	70 (23%)	131 (17%)	
Family of origin	177 (57%)	482 (64%)	
Homeless	1 (0.3%)	3 (0.4%)	
Non-psychiatric facilities	4 (1.3%)	16 (2.1%)	
Living alone	19 (6.2%)	33 (4.4%)	
Unknown	58	117	
Substance use			<0.001
Yes	78 (22%)	410 (48%)	
Unknown	11	25	
DUP			0.014
Mean (SD)	8 (12)	9 (12)	
Unknown	19	67	

a *n* (%).

b Wilcoxon rank-sum test; Fisher’s exact test.

### Effect of socioeconomic factors on FEP incidence

In all instances, multilevel models accounting for municipality and province, including random intercepts and slopes for adjustment variables, performed better than both the BYM2 models and simpler multilevel models with random intercepts only (Table 3). Each analysis were adjusted for confounders identified through the DAG (Supplementary Table 1). Models with cannabis use as the main exposure included only random intercepts, as random slopes did not improve model fit or convergence in this case.

**Table 3.** Effect of socioeconomic factors on FEP incidence and characteristics

Outcome	Exposure	Estimate	Est. Error	l−95% CrI	u−95% CrI	Rhat	Probability coefficient over/below zero^1^	Plot	Average marginal effect^2^
Incidence	Population density	0.10	0.04	0.02	0.19	1.00	99% (+)	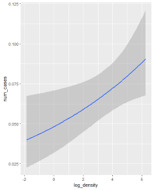	Min–max: 8.4 cases (0.81; 14.7); IRR 2.54 (1.25; 6.60) 1SD: 1.40 cases (0.20; 2.42); IRR 1.14 (1.03; 1.29)
	Educational deprivation	0.13	0.06	0.00	0.26	1.00	98% (+)	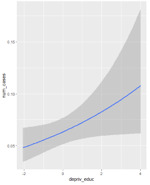	Min–max: 14.2 cases (0.68; 39.41); IRR 3.22 (1.26; 14.3) 1SD: 1.54 cases (0.15; 3.48); IRR 1.15 (1.02; 1.31)
	Area cannabis use	0.15	0.08	−0.01	0.32	1.00	97% (+)	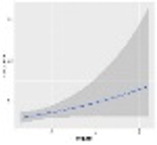	Min–max: 10.39 cases (−0.89; 26.32) IRR 2.34 (0.934; 6.32)1SD: 3.15 cases (−0.29; 6.86) IRR 1.31 (0.98; 1.82)
Migrant (yes/ no)	Economic inequality	5.03	3.12	−1.23	11.10	1.00	95% (+)	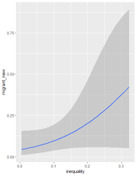	Min–max: 28% (−6%; 59%): OR 3.18 (0.52; 14.5) 1SD: 5% (−1%; 11%); OR 1.28 (0.95; 1.73)
	Migrant density (% migrants)	0.10	0.04	0.03	0.18	1.00	100% (+)	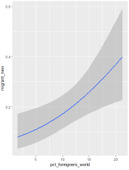	Min–max: 31% (10%; 50%); OR 4.81 (1.00; 23.1) 1SD: 6% (2%; 10%); OR 1.31 (1.08; 1.60)
Substance use (yes/no)	Population density	−0.14	0.07	−0.28	0.01	1.00	97% (-)	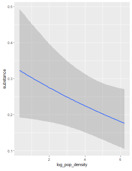	Min–max: − 18% (−35%; 0.7%); OR 0.65 (0.44; 1.08) 1SD: −3% (−7%; 0.2%): OR 0.91 (0.83; 1.00)
	Deprivation	0.09	0.04	0.01	0.18	1.00	98% (+)	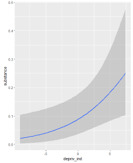	Min–max: 18% (2%; 50%); OR 3.44 (1.21; 21.8) 1SD: 4% (0.3%; 8%); OR 1.12 (1.01; 1.26)
DUP (months)	Migrant density (% migrants)	−0.04	0.02	−0.08	0.00	1.00	97% (-)	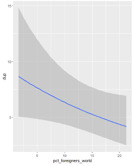	Min–max: − 21.4 months (−7179; 4.16) 1sd: − 3.35 months (−1122; 0.76)
	Area cannabis use	0.18	0.11	−0.03	0.39	1.00	95% (+)	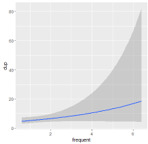	Min–max: 39.6 months (−9.73; 19261) 1SD: 12.9 months (−2.86; 6229)

The table reports the association between each exposure and outcome, only where the exposure had a 95% or higher posterior probability of being non-zero. Each row reports the median parameter value of the exposure (fixed effect), its 95% 95% CrI, Rhat value.

1. The ‘Probability coefficient non-zero’ column reports the posterior probability of direction, i.e. the probability of the hypothesis that the coefficient of the fixed effect is above (+) or below (−) zero. For instance, population density had a 99% posterior probability of being above zero. 2. The column ‘Average Marginal Effect’ reports the marginal effect, i.e. the effect on the outcome for modifications of the exposure. Marginal effects are reported in the response scale (i.e. for incidence: number of cases per 100 000 persons year; for DUP: months; for dichotomous outcomes: probability of ‘yes’), as OR or IRR. They correspond to a change of the predictor from the minimum to maximum value (‘min–max’) or to a change of one standard deviation around the mean value (1SD). Predictors (operational definition): Population density (number of residents per km^2^ (log-transformed); Educational deprivation (% of residents aged 15–60 years with only primary education); Global deprivation (Composite index of social and material disadvantage); Economic inequality (Ratio of population in highest vs lowest income deciles); Migrant density (% of residents born outside Italy); Cannabis use (% of 15- to 19-year-old students reporting ≥ 20 uses in past 30 days in the province); Outcomes: Incidence (new FEP cases per 100 000 at-risk individuals/year); DUP (months elapsing from psychosis emergence to first antipsychotic treatment); Migrant status (FEP case born outside Italy); Substance use (clinically assessed problematic use of any psychoactive substance).

There was a high probability (>95%) that higher population density determined an increase of FEP incidence, so that an increase of population density of the entire range was associated with 10.6 additional cases for females (95% Credible Interval [CrI]: 1.47–18.7) and 22.9 for males (95% CrI: 1.43–40.1). Whereas, an increase of population density of a SD around the mean, there were 1.77 additional cases per 100 000 subjects per year for females (95% CrI: 0.29–3.07), and 3.81 for males (95% CrI: 0.45–6.63). On average, this corresponds to an IRR of 1.14 (95% CrI: 1.03; 1.29).

A change of the entire range of educational deprivation also corresponded to a significant increase of incidence (Table 3, 98% posterior probability): 18.5 cases per 100 000 subjects per year for females (95% CrI: 1.38; 52.78), and of 37.83 males (95% CrI: 1.00; 104). An increase of one standard deviation of educational deprivation was associated with an increase of 2.00 female cases per 100 000 subjects per year (95% CrI: −0.07; 4.46), and 4.11 for males (95% CrI: 0.58; 9.48). On average this corresponds to an IRR of 1.15 (95% CrI: 1.02; 1.31).

There was a high probability (97%) that higher levels of province-level cannabis use determined an increase of FEP incidence, after adjusting for population density, global and educational deprivation, migrant density, gender, and year, so that an increase of the entire range of cannabis use corresponded to a large increase of incidence (Table 3): 17.2 cases per 100 000 subjects per year for females (95% CrI: −1.07; 61.39), and of 23.67 males (95% CrI: −2.51; 44.14). An increase of one standard deviation of province-level cannabis use was associated with an increase of 5.40 female cases per 100 000 subjects per year (95% CrI: −0.35; 16.04), and 7.16 for males (95% CrI: −0.82; 11.54). On average this corresponds to an IRR of 1.31 (95% CrI: 0.98; 1.82).

Increases of the global deprivation index were associated with an increase of incidence, although with a lower level of credibility (ST 2, posterior probability: 89%, IRR: 1.13; 95% CrI: 0.96; 1.34). Whereas, economic inequality and migrant density were not consistently associated with changes of incidence.

### Effect of socioeconomic factors on FEP incidence

**Figure 3.**
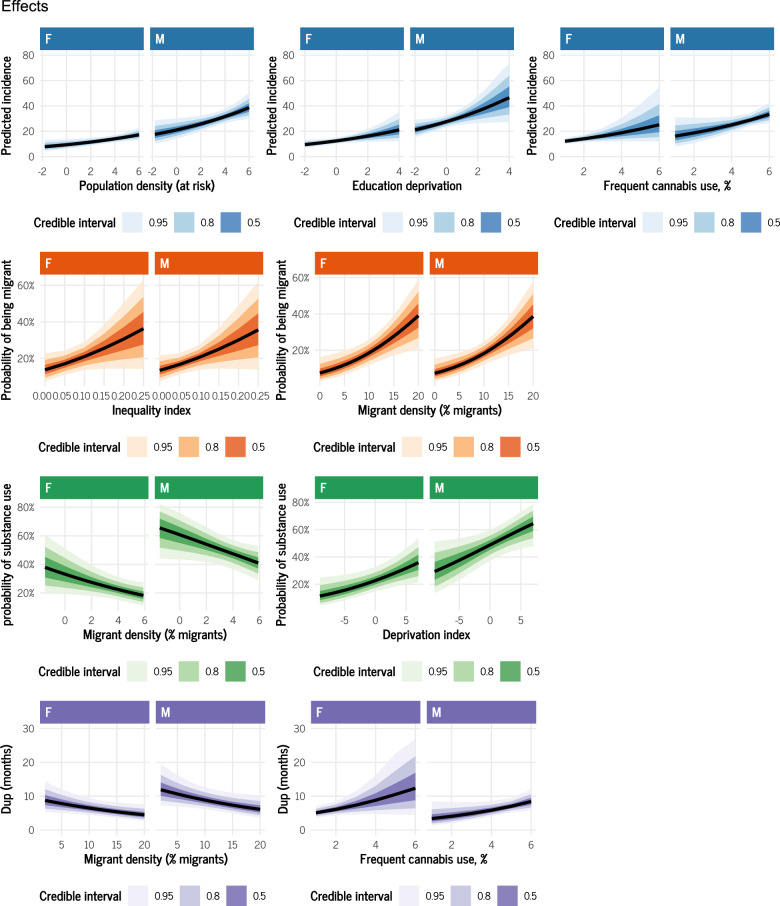
Average Marginal Effects of socioeconomic factors on FEP incidence and characteristics.

Among the most credible effects (Table 3; Fig. 3), economic inequality increased the probability of observing FEP among migrants than among native individuals (OR for a 1SD increase of exposure: 1.28; 95% CrI: 0.95; 1.73). The migrant density in the area was associated with an increase of the probability of observing migrant FEP (OR for a 1sd increase: 1.31; 95% CrI: 1.08; 1.60). Population density was associated with reduced likelihood of substance use (OR for 1sd increase: 0.91; 95% CrI: 0.83; 1.00). Whereas, global deprivation increased the likelihood of using substances (OR: 1.12; 95%CrI: 1.01; 1.26). Finally, the DUP was affected by migrant density in the area, so that a change of a standard deviation increase of the proportion of migrants corresponded to a DUP 3.3 months shorter and by area-level frequent cannabis use, so that a change of the standard deviation of cannabis use increased the DUP of 13 months (95% probability).

## Discussion

This study is one of the few to examine the joint effects of multiple socioeconomic factors on the epidemiology of FEP. In a high-income large European region, with mixed urban and rural settings we found that area-level educational deprivation, population density and frequent cannabis use increased the incidence of psychosis. These, and other socioeconomic factors also influenced treatment delay, the probability of observing FEP cases who used illicit substances, and who were migrants. These findings may allow customization of care resources and components to meet the specific needs of the populations with firstFEP.

Our results are largely consistent with those of other recent studies detecting an effect of population density across the rural–urban gradient, and of area-level cannabis use on the incidence of FEP (Brink *et al*., 2024; Grover *et al*., 2024; Kirkbride *et al*., 2017; McDonald *et al*., 2021). Whereas, unlike other reports, in our study the effect of educational deprivation was stronger than those of economic deprivation. The effect of education is unlikely to depend on differences in the DUP or substance use among cases, as we did not detect associations between education deprivation and these outcomes. Emilia-Romagna has a relatively high income and low educational attainment compared with other European regions, and these two indices were weakly correlated in our study, unlike in others (Sirin, 2005). Thus, the effect of education may have emerged because it reflects other social factors than economic ones, which elevate psychosis risk. Educational deprivation may increase FEP incidence through various mechanisms. First, areas with lower educational levels may be characterized by lower mental health literacy, higher discrimination and higher social pressure towards individuals at risk for psychosis, potentially hastening its onset (Belvederi Murri *et al*., 2016; Sum *et al*., 2022; Takizawa *et al*., 2021). Second, educational deprivation may be linked with worse cognitive performance, as has been recently shown (Lewis *et al*., 2020). In the study by Lewis and colleagues, this effect was deemed independent from genetic factors (Lewis *et al*., 2020). Arguably, societal and individual educational deprivation may lead to worse inferential abilities and more frequent cognitive biases (Corlett and Fletcher, 2021) that might increase psychosis risk. Lastly, individuals with lower education may be subject to overdiagnosis of psychosis because of cultural bias (Morgan *et al*., 2019).

We identified an association between area-level cannabis use and psychosis incidence, which was not limited to affective psychoses, unlike in recent analyses of the EU-GEI study (Brink *et al*., 2024). However, direct comparisons are limited by differences in study design, the operationalization of cannabis exposure, and the geographic scale of analysis—including variation in urban/rural composition. These methodological and contextual distinctions likely contribute to the divergent findings and underscore the need for further research on how cannabis use and local sociodemographic environments interact in shaping psychosis risk (Belvederi Murri *et al*., 2025; Brink *et al*., 2024).

This study is one of the few, to our knowledge, to link area-level socioeconomic factors with individual characteristics. Few other reports suggest that population density, ethnic composition and income inequality may not only affect incidence rates, but also differences in the clinical presentation (Tibber *et al*., 2019) and, in some cases, clinical outcomes (Griffiths *et al*., 2023). In our study, individuals with FEP were more likely to have engaged in substance use if they came from areas with higher deprivation and decreasing population density. The lower prevalence of substance use within high-density areas is different from findings of other studies (Morgan and Mall, 2019; Schifano, 2008), and confirms that the effect of socioeconomic risk factors may present specific patterns and modalities across different settings. In Emilia-Romagna higher-density municipalities are generally wealthy, socially connected, and may offer greater access to health education and preventive services, potentially leading to healthier lifestyles and less harmful use patterns (Belvederi Murri *et al*., 2023; Brink *et al*., 2024).

Cannabis use among FEP individuals commonly represent an attempt at self-medication, and requires targeted care strategies, similar to the tailored management required for FEP patients who are either first- or second-generation migrants (Tarricone *et al*., 2021) or domestic migrants (Tarricone *et al*., 2016). This study also suggests that mental health services in areas with greater economic disparity should be specifically equipped to care for cases of FEP who are migrants (D’Andrea *et al*., 2023). Finally, the DUP was increased by area-level cannabis use, and decreased by higher area-level density of migrants. Of note, associations with factors at the area-level should be interpreted differently from those detected at the individual level, as in other studies (Hastrup *et al*., 2018). Effects of area-level features may reflect differences in the societal appraisal of symptoms. In areas with high cannabis use, behavioural symptoms of psychosis may be more frequently normalized and help-seeking delayed, or may be more frequently linked with paranoid or avoidant psychopathology (Belvederi Murri *et al*., 2025). In areas with higher migrant density, protective effects of lower stigmatization and better social cohesion may lead to earlier detection of psychosis (Anglin *et al*., 2023; Morgan *et al*., 2019; Veling *et al*., 2008).

### Strengths and limitations

The large sample size and long observation period, over a region with mixed urban and rural area suggests that study results are representative of high-income populations of FEP seen in clinical services. Exposures include a comprehensive array of environmental factors that were measured prospectively, except for deprivation indices. Coupled with a rigorous approach to analyses, our study ought to reduce bias due to confounding and provide reliable estimates of total assumed causal effects (McElreath, 2018).

Limitations include a possible underestimation of incidence (e.g. patients entirely seen within the private sector), despite the consolidated, proactive methodology for early detection (Belvederi Murri *et al*., 2021). Information on some exposures or confounders were not available, such as detailed ethnic composition, social cohesion, type of cannabis availability, use in other age groups, or other detailed data on cannabis use (e.g. potency), as well as other substance use (Di Forti *et al*., 2019), diagnosis, individual income or familial characteristics – this may have prevented us from detecting some associations with sufficient level of confidence. While clinicians used a shared, standardized definition of DUP, we did not include a standardized rating instrument and did not collect IRR data. Finally, we did not directly consider hypotheses related to reverse-causation, namely the link between genetic vulnerability and drift towards selected social contexts (Logeswaran *et al*., 2023; Maxwell *et al*., 2021). Our DAG reflects a contextual (structural) perspective, where population density shapes the sociodemographic composition of areas. To account for the possibility of compositional confounding, we ran a sensitivity analysis for the density–incidence association, including mean age of FEP and proportion of migrants among FEP cases in each municipality. The results showed sign reversal and poor convergence, potentially indicating a collider structure. Given the rarity of FEP and the high number of municipalities with zero cases, our ability to isolate contextual effects may have been limited. These results do not exclude that compositional mechanisms may also contribute, and further research integrating both perspectives may help to clarify how population structure and area-level factors interact to influence psychosis risk.

### Clinical relevance

This study, supporting the ability to predict incidence and characteristics, enables resource allocation, tailoring of clinical services for FEP based on service user needs (McDonald *et al*., 2021), clinical prediction or support to decisions re. policy. In addition, it may help to target awareness campaigns on psychosis, which might reduce the DUP (Connor *et al*., 2016) or improve stigma attitudes and help seeking (Belvederi Murri and Amore, 2018; Xu *et al*., 2018).

In conclusion, we found that the incidence of psychosis was influenced by population density, low population education levels and cannabis use. Area-level deprivation, economic inequality, cannabis use and migrant density influenced the DUP and other individual features of patients with FEP presenting in public mental healthcare services.

## Supporting information

10.1017/S2045796025100206.sm001 Belvederi Murri et al. supplementary material Belvederi Murri et al. supplementary material

## Data Availability

Data are not publicly available, due to inability of the funding agency to share healthcare data with third parties.

## Supplementary material

The supplementary material for this article can be found at https://doi.org/10.1017/S2045796025100206.
