# Supplementary material for: The impact of socioeconomic factors on the incidence and characteristics of first-episode psychosis
Source: Epidemiol Psychiatr Sci. 2025 Sep 2;34:e45. doi: 10.1017/S2045796025100206 (PMC12450539; doi:10.1017/S2045796025100206)
Supplement: Belvederi Murri et al. supplementary material [file S2045796025100206sup001.docx]

Supplementary material for

**Socioeconomic factors and first-episode psychosis in a high-income European region**

Summary

[S1. Supplementary methods 2](#_Toc194995737)

[S1.1 Data collection 2](#_Toc194995738)

[S1.2 Spatial data and information on predictors 2](#_Toc194995739)

[S1.3 Data preparation 3](#_Toc194995740)

[S1.4 Directed Acyclic Graphs 3](#_Toc194995741)

[S1.5 Bayesian multilevel models 4](#_Toc194995742)

[S2. Supplementary results 4](#_Toc194995743)

[S2.1 Literature review 4](#_Toc194995744)

[S2.1.1 Urbanicity, density 4](#_Toc194995745)

[S2.1.2 Area level deprivation 4](#_Toc194995746)

[S2.1.3 Area level inequality 5](#_Toc194995747)

[S2.1.4 Area level ethnic background, cultural and educational factors 5](#_Toc194995748)

[S2.1.5 Individual, familial economic or relational problems / adverse childhood experiences 5](#_Toc194995749)

[S2.2 Development of the Directed Acyclic Graph 6](#_Toc194995750)

[SF 1. Analysis of HoNOS ratings 7](#_Toc194995751)

[SF 2. Characteristics of individuals with First Episode Psychosis in Emilia-Romagna 8](#_Toc194995752)

[SF 3. Correlation between sociodemographic indices 9](#_Toc194995753)

[SF 4. Sociodemographic indices: population density 10](#_Toc194995754)

[SF 5. Sociodemographic indices: global deprivation index 11](#_Toc194995755)

[SF 6. Sociodemographic indices: educational deprivation index 12](#_Toc194995756)

[SF 7. Sociodemographic indices: inequality index 13](#_Toc194995757)

[SF 8. Sociodemographic indices: migrant density (proportion of migrants) 14](#_Toc194995758)

[SF 9. Correlation between global deprivation index and educational deprivation index 15](#_Toc194995759)

[SF 10. Correlation between global deprivation index and inequality index 16](#_Toc194995760)

[SF 11. Correlation between global deprivation index and migrant density 17](#_Toc194995761)

[SF 12. Spatial distribution of cases of First Episode Psychosis over 7 years in Emilia-Romagna 18](#_Toc194995762)

[ST 1. Model adjustment sets provided by DAG analysis 19](#_Toc194995763)

[ST 2. Associations between exposures and outcomes: incidence 21](#_Toc194995764)

[ST 3. Associations between exposures and outcomes: age at onset 22](#_Toc194995765)

[ST 4. Associations between exposures and outcomes: migrant status 23](#_Toc194995766)

[ST 5 Associations between exposures and outcomes: unemployment 24](#_Toc194995767)

[ST 6. Associations between exposures and outcomes: substance use 25](#_Toc194995768)

[ST 7. Associations between exposures and outcomes: DUP 26](#_Toc194995769)

[ST 8. Associations between exposures and outcomes: Relational problems (HoNOS item 9) 27](#_Toc194995770)

[ST 9. Associations between exposures and outcomes: resource problems (HoNOS item 11 and 12) 28](#_Toc194995771)

[ST 10. Summary of the associations with 80% or higher posterior probability of direction 29](#_Toc194995772)

[Supplementary References 32](#_Toc194995773)

# S1. Supplementary methods

## S1.1 Data collection

The RER-FEP database was developed and maintained by the coordinating centre of Modena (Ferrara et al. 2019). Data was linked with those from the regional administrative database from the Emilia-Romagna regional health office, allowing to verify the potential presence of additional subjects who had not been screened for the FEP program, to validate sociodemographic data (e.g. country of birth, municipality of residence). The RER-FEP database contains sociodemographic and clinical assessments collected by clinicians from out- and inpatient centres across local Health Trusts of nine provinces (Bologna, Ferrara, Forlì-Cesena, Modena, Parma, Piacenza, Ravenna, Reggio Emilia, Rimini).

The HoNOS data comprises 12 items assessing behaviour (item 1 – 3), impairment (4 – 5), mental symptoms (6 – 8) and social functioning (9 – 12) in the period preceding first contact with mental health services (Lora et al. 2001). Each Mental Health Department held regular meetings of the FEP team in order to coordinate clinical action and assessments, with yearly training sessions to improve inter-rater reliability.

## S1.2 Spatial data and information on predictors

Geographical data for the Emilia-Romagna region was retrieved from the 1.4.1 *mapping* R package (Serafini e Ferrara 2023). Data is relative to 2018, where the region was divided into 331 municipalities. Adjustment were made to accommodate few instances where municipalities were joined over the study period. In such cases, the average of joined municipalities characteristics was used for the resulting one. Population density per each study year was calculated as the total number of inhabitants per square kilometre, and log-transformed.

Information on deprivation was obtained from the Istat 2011 census (ISTAT Italian Institute of Statistics 2020). We followed the protocol of a recent study which used a range of deprivation indices from the social, economic, housing and education domains at the level of municipality to elaborate a global deprivation index (Caranci et al. 2010; Rosano et al. 2020). The global deprivation index was derived from: 1) percentage of the population aged 15-60 having elementary education or less (henceforth “educational deprivation”); 2) percentage of the population unemployed or seeking first employment; 3) mean house occupancy per 100 square meters; 4) percentage of people living in rented accommodation; 5) proportion of single-parent households with underage children. All indices were transformed into z-scores. In preliminary analyses, the educational deprivation index was less strongly correlated with other indices and with the global deprivation index (R = 0.283, other indices all R > 0.600). Therefore, we examined the role of educational deprivation in addition to the global deprivation index.

The economic inequality index was computed based on the top income share principle (OECD Data 2024; Piketty e Saez 2014), specifically the ratio between the percentage of subjects in the top two income brackets (75 thousand to 120 thousand euros; 120 thousand euros per year, and over) divided by the percentage of subjects in the lowest bracket (less than 10 thousand euro per year).

The presence of migrants in each municipality was expressed as the proportion of persons born outside Italy over the total population, per each year. This was interpreted as an index of migrant density (Boydell et al. 2001). Data was retrieved from the Italian national census (ISTAT Italian Institute of Statistics 2020).

Data on cannabis use was derived from the ESPAD 2019 census (Benedetti et al. 2021). The survey assessed the use of cannabis use in high schoolers among 15 and 19 years of age, at the provincial level. Among different indices (use in lifetime, last year, last month) we adopted the percentage of frequent cannabis use, that is, having reported use in the last 30 days with a frequency equal or higher than 20 times. Frequency of cannabis use has been identified as a contributor to the variability of FEP incidence (Di Forti et al. 2019).

## S1.3 Data preparation

To examine the effect of socioeconomic factors on incidence, the unit of analysis was established at the level of the 331 municipalities from the nine provinces of the Emilia-Romagna region. To examine the effect of socioeconomic factors on individual characteristics, the unit of analysis was established at the participant level, matched with the socioeconomic data from the corresponding municipality of residence in the year of psychosis onset (except for deprivation indices, which was constant across years).

To analyse the impact of socioeconomic factors on individual social functioning and resource availability we used HoNOS clinical ratings (items 9-12). We first examined the groupings of HoNOS items using network analyses, and the reciprocal relationship of the underlying latent clinical dimensions. We explored the HoNOS data generating mechanism and the dimensionality of data of the 12 items, focusing on those relative to individual problems (item 9 -12). First, we used the Loadings Comparison Test to infer whether clinical data was more likely to be generated from a network structure (assuming mutual causality of individual traits measured by items) or from a latent variable structure (assuming latent factor(s) acting as common cause of interchangeable indicators) (Belvederi Murri et al. 2022; van Bork et al. 2021; Golino e Christensen 2020). According to the more probable data generating mechanism, we reduced data using factor or network analyses plus hierarchical Exploratory Graph Analysis to identify the latent clinical traits (Belvederi Murri et al. 2022; Samo et al. 2022). This strategy has the advantage of reducing the impact of measurement error.

## S1.4 Directed Acyclic Graphs

We employed a causal inference approach to the research questions (Lipsky e Greenland 2022). This method aims to reduce the risk of bias when estimating the causal effect of exposures (in this case, socioeconomic factors) on outcomes (in this study, FEP incidence and individual characteristics). We used Directed Acyclic Graphs (DAGs) to exemplify and illustrate our causal assumptions transparently and a-priori. This approach has two important advantages: (1) DAGs allow other researchers to evaluate the underlying causal model in detail, and propose alternative hypotheses. For this purpose, we have included a link to a modifiable versions of the DAG in the figure legend, so that readers can each assumption and the impact of changes to the assumed causal structure; (2) DAGs allow to determine which variables represent confounders, colliders and/or mediating variables in each analysis. This point is particularly relevant for our study, since it comprises a high number of exposure and outcome variables. In such cases it would be incorrect to perform a single multivariable model (e.g. deprivation, population density and migrant density as exposures, FEP incidence as outcome) and interpret each exposure coefficient as an estimate of its causal effect on the outcome (Westreich e Greenland 2013). Whereas, the DAG is analysed with logic rules to determine which variables function as confounders, colliders, mediators (or neither) *for each couple of exposure and outcome*. Thus, we obtain the minimal adjustment set that is more appropriate for each separate model (e.g. the model of the effect of deprivation on incidence, the model of the effect of population density on incidence, and so on). Of note, we sought to identify the total effect of each exposure on each outcome, thus identified adjustment sets generally do not include mediating variables.

To build the DAG, three researchers (MBM, AO, CP) performed a literature review using pertinent keywords (psychosis, schizophrenia, first-episode psychosis, deprivation, inequality, economic, population density, cannabis, spatial analysis). We sought to identify literature that examined the relationships between socioeconomic factors, first episode psychosis incidence and characteristics. We selected literature reviews and studies with high methodological quality (e.g. comprehensive approach to socioeconomic factors, accounting for spatial distribution). We were also interested in identifying causal pathways or plausible mechanisms linking them (e.g. putative mediators). Given the above considerations on the “Table 2 fallacy” (Westreich e Greenland 2013), and considering the notable geographic heterogeneity of sociodemographic risk factors (Jongsma et al. 2018, 2019; Kirkbride et al. 2024), we adopted an inclusive approach for the inclusion of candidate causal links, rather than considering only nominally significant associations. If disagreements developed re. the role of a variable (e.g. economic deprivation causing economic inequality or vice-versa), we solved it by discussion, while considering the level and timeframe of measurement of each variable. As a necessary approximation, we did not explicitly account for the role of genetic factors. The DAGs were elaborated with the *dagitty* web application (<https://dagitty.net/>) and the corresponding R software package (Textor, Hardt, e Knüppel 2011).

## S1.5 Bayesian multilevel models

In a preliminary analysis, we used the *geostan* package to explore whether FEP incidence was characterized by meaningful spatial autocorrelation, which is common working with spatial data (Donegan 2022). The relationship between each exposure and incidence was analysed using models of increasing complexity, adjusting for gender, year and the minimal sufficient set of confounders identified by the DAG (ST 2). We compared: 1) a simpler hierarchical model, where incidence was nested only within municipalities; 2) a hierarchical two-level model where municipalities were nested within provinces. This would account for potential variability due to further differences between each local healthcare agency in the detection of FEP; 3) a modified Besag York Mollié (BYM2) model that accounts for both space- and time- related dependencies and autocorrelation (Morris et al. 2019; Riebler et al. 2016). If simpler models converged, we also: 1) allowed the intercept and the slopes of predictors to vary by municipality and/or province introducing them as random effects; 2) tested a location-scale model where also the *variance* of incidence was allowed to vary across municipalities (i.e. with a “*shape* ~ 1 + (1 | municipality)” formula); 3) tested whether the density of larger cities (provincial capitals and others) might have distinct predictive value that would improve the models, by adding an interaction term between socioeconomic predictors and population density. We used out-of-sample predictive accuracy using Leave-One-Out Cross Validation (LOO-CV) to compare model accuracy and select the final model to report (Bürkner, Gabry, e Vehtari 2021). Models were run on four chains, with 7000 iterations each (1000 discarded) using default uninformative priors. Model were checked for convergence using conventional indices and visually.

The analysis of the relationship between socioeconomic factors and FEP characteristics was based on a similar analytic approach, although we did not test spatial autocorrelation and interactions. Here, outcome distributions were based on lognormal (age of onset and DUP), cumulative (HoNOS items of family and resource problems) and Bernoulli (substance use, unemployment, migrant status) distributions.

# S2. Supplementary results

## S2.1 Literature review

Here, we summarise the main findings of the literature review on the association between socioeconomic factors, FEP incidence and characteristics.

### S2.1.1 Urbanicity, density

Systematic reviews of studies examining the correlation between urbanicity and psychosis confirm the link between urbanicity (urban birth or upbringing, urban living), and psychotic disorders (Grover, Varadharajan, e Venu 2024; March et al. 2008). The association seems highly variable across geographic areas, and might depend on other factors such as area-level deprivation, migrant density and social fragmentation (Grover et al. 2024). Relevant hypotheses in this regard are social causation, social drift or social selection processes (Kirkbride, Hameed et al. 2017).

### S2.1.2 Area level deprivation

Socioeconomic disadvantage, especially when encountered in neurodevelopmental age, is associated with worse mental health outcomes (Kirkbride et al. 2024; Reiss 2013). More specifically for psychosis, a review of 28 studies found a strong and highly replicated association between social deprivation at birth and higher risk of psychotic disorders (O’Donoghue, Roche, e Lane 2016). Recently a study in turkey found a relationship between indices of economic disadvantage and the onset and persistence of psychotic experiences, and with the progression along the extended psychosis phenotype (Binbay, Erel et al. 2023).

The explanations for this finding include both neighbourhood-level characteristics (weaker social cohesion, higher crime rates, higher availability of illicit drugs) and individual characteristics (higher stress due to greater family problems and frequent residential mobility). A subsequent study showed that deprivation during upbringing was correlated with increased risk of serious mental illness using a rigorous methodology (Logeswaran, Dykxhoorn et al. 2023), similarly to the series of reports from the East Anglia SEPEA study (Kirkbride, Jones et al. 2014, Kirkbride, Stochl et al. 2015, Kirkbride, Hameed et al. 2017). Area-level deprivation might cause an increase of psychosis incidence also by increasing illicit drugs availability and use in the population (Morgan e Mall 2019). We hypothesized that area level deprivation might specifically influence the likelihood of observing greater economic problems among participants and/or relational problems. Finally, more deprived areas may be characterized by relative lack of resources for mental health services (in part due to greater demands by the population) and possible cultural factors which might result in different referral systems a longer DUP (Schultze-Lutter et al. 2015). In a recent study this was found to be independent of demographic factors (Reichert e Jacobs 2018). Similarly, economic prosperity at the national level has been linked with notable differences in the DUP (Large et al. 2008). Finally, deprivation has been linked with differences in psychotic symptom severity or profiles (e.g. paranoia, but not hallucinations or hypomania) (Wickham, Taylor et al. 2014).

### S2.1.3 Area level inequality

Income inequality is linearly associated with higher rates of mental illness in high income countries (Marbin et al. 2022; Pickett e Wilkinson 2010; Tibber et al. 2022). Considering psychosis, incidence rates has been found to be significantly higher in areas with higher income inequality, even accounting for indices of multiple deprivation in the East Anglia study, which analysed data at the neighbour level (Kirkbride, Jones et al. 2014, Kirkbride, Hameed et al. 2017). Data from the Welsh Health Survey showed, however, a complex pattern of association between income inequality and the prevalence of common mental disorders at different area level of analysis, so that: 1) within neighbourhoods with *low* deprivation, income inequality was associated with *better* mental health. Income inequality, of note, was inversely correlated with deprivation at the neighbour level; 2) the positive relationship between inequality and mental disorders was drastically reduced after adjusting for overall economic deprivation (income); 3) at the larger regional level, *higher* inequality was associated with significantly *poorer* mental health, in contrast to findings at the neighbourhood level (Fone, Greene et al. 2013). This stresses the need to account for multiple levels of analysis and for both deprivation and inequality.

No significant association was detected between inequality and cannabis use in a study from Germany (Henkel and Zemlin 2016) or with substance use in general (Morgan e Mall 2019) However, there is limited research on the subject. We also found limited research on the effect of inequality on other individual risk factors for psychosis.

### S2.1.4 Area level ethnic background, cultural and educational factors

The role of stigma and mental health literacy

Lower neighbourhood education was correlated with higher risk of **Relational problems** such as child maltreatment and intimate partner violence (Gracia, López-Quílez et al. 2018). In general, higher **education** is bidirectionally associated with better mental health, reductions in suicide attempts (Rosoff et al. 2020).

Relational problems cause lower education (Myhr, Lillefjell et al. 2017). Individual education is higher in higher education families (Myhr, Lillefjell et al. 2017), no paper about correlation between area level (family=proxy?) and individual level education. We found no studies about the association between area level education and individual economic problems, educational attainment or individual mental health disorders. However, people from low education neighbourhoods had longer referral times to see a psychiatrist (Steele, Glazier et al. 2009). Lower area-level education was correlated with higher odds of alcohol use, but lower average daily alcohol consumption (Lê, Ahern et al. 2010). Marijuana use was higher in areas with higher education inequality (Galea, Ahern et al. 2007).

### S2.1.5 Individual, familial economic or relational problems / adverse childhood experiences

There is abundant literature on individual social factors, such as loneliness, social isolation, and Adverse Childhood Experiences (ACEs) influencing psychosis incidence (Kirkbride et al. 2024; Stilo e Murray 2019) – in particular loneliness, perceived social support, and social network characteristics are deemed to influence psychosis risk (Pearce et al. 2023).

Considering economic difficulties, the 2010 Australian national survey of psychotic disorders (SHIP) found that 85% of patients with psychosis rely on a government benefit as their main source of income. Only 33% have any paid work over the course of a year (Morgan, Waterreus et al. 2017). The direction of this association however, is still debated, i.e. whether social causation or social drift/ social selection mainly drive this phenomenon. Recent evidence suggest that social causation may be particularly relevant for the development of psychosis among individuals living in deprived areas, but reverse causation pathways (e.g. social immobility) seems also relevant (Logeswaran et al. 2023). It remains to be established whether area-level deprivation causes an increase in psychosis risk by worsening individual economic resources.

Other relevant pathways for the effects of disadvantaged socioeconomic conditions are increases of childhood maltreatment, which was also related to poorer treatment outcomes in psychotic disorders (Thomas, Höfler et al. 2019). For instance, violence in the home context was found to be a predictor of psychosis, as part of cumulative traumas that lead augmented risk of psychosis (Shevlin, Houston et al. 2008). It may also have effects of anticipating the age at onset (Comacchio, Howard, et al. 2019; Comacchio, Lasalvia, e Ruggeri 2019). A proxy for ACEs and traumatic experiences might by poor family support or relational problems (Georgiades, Almuqrin et al. 2023).

Finally, cultural factors, such as area-level, family-level, or individual education may be linked with worse mental health literacy, and stigma or discriminative behaviour against individuals with mental health conditions (Eliasson et al. 2021; Sarraf, Lepage, e Sauvé 2022; Sum et al. 2024), lower family resiliency and lower family awareness of mental illness can be associated with insight related to the illness, treatment acceptance (Belvederi Murri e Amore 2018; Kudva et al. 2020) and with a longer treatment delay (Paquin-Goulet, Krishnadas et al. 2023).

## S2.2 Development of the Directed Acyclic Graph

The following figure is the DAG used to illustrate the causal relationships relative to the incidence and characteristics of First Episode Psychosis in the population exposed to area-level sociodemographic and economic factors. The DAG encodes a set of causal assumptions between variables of interest. The red circles represent the area-level exposures (population density, socioeconomic deprivation, educational deprivation, area-level frequent cannabis use, migrant density, economic inequality). The blue circles represent the outcomes of the study (area incidence of FEP, age of onset, migrant status, unemployment, substance use, DUP, relational/family problems, resource problems). The yellow circles are other contextual and individual variables. The grey circles are unmeasured variables (resources of the Mental Health system, stigma towards mental health conditions). A manipulatable version of this DAG is available at <https://dagitty.net/mLqheYnQr>. Users can select different variables as the exposure and outcome to receive information on the minimal adjustment set of confounders in each analysis (reported in ST 3).

### SF 1. Analysis of HoNOS ratings

The Network Analysis of HoNOS ratings (total of 12 items) revealed a more probable Network structure than a latent factor structure (Loadings Comparison Test: Factor: 16%, Network: 84%). The hierarchical Exploratory Graph Analysis with Louvain algorithm suggested that HoNOS data was organized into five lower-order communities and one higher-order community. The items of interest (#9, #11, #12) were distributed as follows: item #9 (interpersonal problems) in community 4 with items 10 and 8; item #11 and #12 (Living Conditions and Work and Leisure resource problems) in community 5. Thus, we analysed item #9 as a measure of interpersonal/relational problems, and a composite of item #11 and #12 as a measure of resource problems.

### SF 2. Characteristics of individuals with First Episode Psychosis in Emilia-Romagna

SF 3. Correlation between sociodemographic indices****

SF 4. Sociodemographic indices: population density

### SF 5. Sociodemographic indices: global deprivation index

### SF 6. Sociodemographic indices: educational deprivation index

### SF 7. Sociodemographic indices: inequality index

### SF 8. Sociodemographic indices: migrant density (proportion of migrants)

### SF 9. Correlation between global deprivation index and educational deprivation index

SF 10. Correlation between global deprivation index and inequality index

SF 11. Correlation between global deprivation index and migrant density

### SF 12. Spatial distribution of cases of First Episode Psychosis over 7 years in Emilia-Romagna

### ST 1. Model adjustment sets provided by DAG analysis

| Outcome | Exposure | Minimal Adjustment Set(s) | Additional variables for stratification | Final predictors in the model |
| --- | --- | --- | --- | --- |
| Incidence | Population density | **-** | gender, year | **density** + gender + year |
|  | Deprivation | { density } | gender, year | deprivation + density + gender + year |
|  | Deprivation education | { density, deprivation, migrant densitymigrant density } | gender, year | depriv_education + density + deprivation + migrant densitymigrant density + gender + year |
|  | Economic inequality | { density, deprivation, migrant densitymigrant density } | gender, year | inequality + density + deprivation + migrant densitymigrant density + gender + year |
|  | Migrant density (% migrants) | { density, deprivation } | gender, year | migrant densitymigrant density + density + deprivation + gender + year |
|  | Area-level cannabis use | { density, depriv_education, deprivation, migrant densitymigrant density } | gender, year | density, depriv_education, deprivation, migrant density, gender, year |
|  |  |  |  |  |
| Age at onset | Population density | **-** | gender, year | density + gender + year |
|  | Deprivation | { density } | gender, year | deprivation + density + gender + year |
|  | Deprivation education | **{ density, deprivation, migrant densitymigrant density } ***  { deprivation, inequality, migrant densitymigrant density } | gender, year | depriv_education + density + deprivation + migrant densitymigrant density + gender + year |
|  | Economic inequality | **{ density, deprivation, migrant densitymigrant density } ***  { depriv_education, deprivation, migrant densitymigrant density } | gender, year | inequality + density + deprivation + migrant densitymigrant density + gender + year |
|  | Migrant density (% migrants) | **{ density, deprivation }** | gender, year | migrant densitymigrant density + density + deprivation + gender + year |
|  | Area-level cannabis use | **{ density, depriv_education, deprivation, migrant densitymigrant density }**  { depriv_education, deprivation, inequality, migrant densitymigrant density } | gender, year | density, depriv_education, deprivation, migrant densitymigrant density |
|  |  |  |  |  |
| Migrant | Population density | **-** | gender, year | density + gender + year |
|  | Deprivation | { density } | gender, year | deprivation + density + gender + year |
|  | Deprivation education | **{ density, deprivation, migrant densitymigrant density }**  { deprivation, inequality, migrant densitymigrant density } | gender, year | depriv_education + density + deprivation + migrant densitymigrant density + gender + year |
|  | Economic inequality | { density, deprivation, migrant densitymigrant density } | gender, year | inequality + density + deprivation + migrant densitymigrant density + gender + year |
|  | Migrant density (% migrants) | { density, deprivation } | gender, year | migrant densitymigrant density + density + deprivation + gender + year |
|  | Area-level cannabis use | { deprivation, inequality, migrant densitymigrant density }  { density, deprivation, migrant densitymigrant density } | gender, year | + gender, year |
|  |  |  |  |  |
| Unemployment | Population density | **-** | gender, year | density + gender + year |
|  | Deprivation | Density | gender, year | deprivation + density + gender + year |
|  | Deprivation education | **{ density, deprivation, migrant densitymigrant density }** | gender, year | depriv_education + density + deprivation + migrant densitymigrant density + gender + year |
|  | Economic inequality | { density, deprivation, migrant densitymigrant density } | gender, year | inequality + density + deprivation + migrant densitymigrant density + gender + year |
|  | Migrant density (% migrants) | { density, deprivation } | gender, year | migrant densitymigrant density + density + deprivation + gender + year |
|  | Area-level cannabis use | { density, depriv_education, deprivation, migrant densitymigrant density } | gender, year | density, depriv_education, deprivation, migrant densitymigrant density |
|  |  |  |  |  |
| Substance use | Population density | **-** | gender, year | density + gender + year |
|  | Deprivation | Density | gender, year | deprivation + density + gender + year |
|  | Deprivation education | **{ density, deprivation, migrant densitymigrant density }**  { deprivation, inequality, migrant densitymigrant density } | gender, year | depriv_education + density + deprivation + migrant densitymigrant density + gender + year |
|  | Economic inequality | **{ density, deprivation, migrant densitymigrant density }**  { depriv_education, deprivation, migrant density } | gender, year | inequality + density + deprivation + migrant density + gender + year |
|  | Migrant density (% migrants) | { density, deprivation } | gender, year | migrant density + density + deprivation + gender + year |
|  | Area-level cannabis use | **{ density, depriv_education, deprivation, migrant density }**  { depriv_education, deprivation, inequality, migrant density } | gender, year | density, depriv_education, deprivation, migrant density + gender, year |
|  |  |  |  |  |
|  |  |  |  |  |
| DUP | Population density | **-** | gender, year | density + gender + year |
|  | Deprivation | { density } | gender, year | deprivation + density + gender + year |
|  | Deprivation education | { density, deprivation, migrant density }  { deprivation, inequality, migrant density } | gender, year | depriv_education + density + deprivation + migrant density + gender + year |
|  | Economic inequality | {deprivation, migrant density } | gender, year | inequality + density + deprivation + migrant density + gender + year |
|  | Migrant density (% migrants) | { density, deprivation } | gender, year | migrant density + density + deprivation + gender + year |
|  | Area-level cannabis use | { density, depriv_education, deprivation, migrant density } |  | density, depriv_education, deprivation, migrant density |
|  |  |  |  |  |
| Relational problems | Population density | **-** | gender, year | density + gender + year |
|  | Deprivation | Density | gender, year | deprivation + density + gender + year |
|  | Deprivation education | **{ density, deprivation, migrant density }**  { deprivation, inequality } | gender, year | depriv_education + density + deprivation + migrant density + gender + year |
|  | Economic inequality | **{ density, deprivation, migrant density }**  { depriv_education, deprivation } | gender, year | inequality + density + deprivation + migrant density + gender + year |
|  | Migrant density (% migrants) | { density, deprivation } | gender, year | migrant density + density + deprivation + gender + year |
|  | Area-level cannabis use | **{ density, depriv_education, deprivation, migrant density }**  { deprivation, inequality, resource_problems }  { depriv_education, deprivation, inequality } | gender, year | density, depriv_education, deprivation, migrant density + gender, year |
|  |  |  |  |  |
| Resource problems | Population density | **-** | gender, year | density + gender + year |
|  | Deprivation | Density | gender, year | deprivation + density + gender + year |
|  | Deprivation education | **{ density, deprivation, migrant density }**  { deprivation, inequality } | gender, year | depriv_education + density + deprivation + migrant density + gender + year |
|  | Economic inequality | **{ density, deprivation, migrant density }**  { depriv_education, deprivation } | gender, year | inequality + density + deprivation + migrant density + gender + year |
|  | Migrant density (% migrants) | { density, deprivation } | gender, year | migrant density + density + deprivation + gender + year |
|  | Area-level cannabis use | **{ density, depriv_education, deprivation, migrant density }**  { depriv_education, deprivation, inequality } | gender, year | density, depriv_education, deprivation, migrant density + gender, year |
|  |  |  |  |  |

### ST 2. Associations between exposures and outcomes: incidence

| Outcome | Exposure | ML vs BYM2 | Estimate | Est.Error | l-95% CI | u-95% CI | Rhat | Bulk_ ESS | Tail_ ESS | Probability coefficient non-zero | Conditional effect |
| --- | --- | --- | --- | --- | --- | --- | --- | --- | --- | --- | --- |
| Incidence | **Population density** | **ML** | **0.10** | **0.04** | **0.02** | **0.19** | **1.00** | **17795** | **15054** | **99% (+)** | 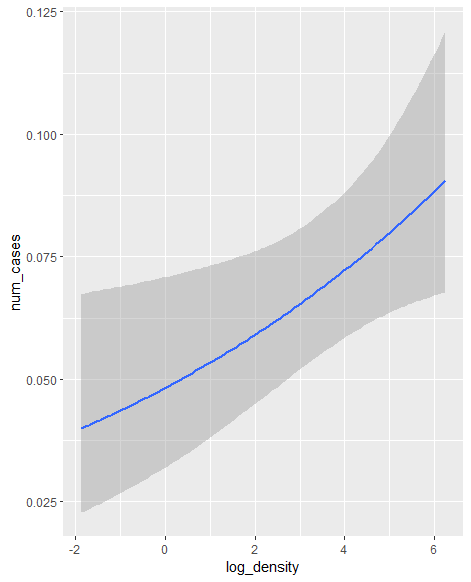 |
| Incidence | Deprivation index | ML | 0.03 | 0.03 | -0.02 | 0.08 | 1.00 | 16027 | 15201 | 89% (+) | 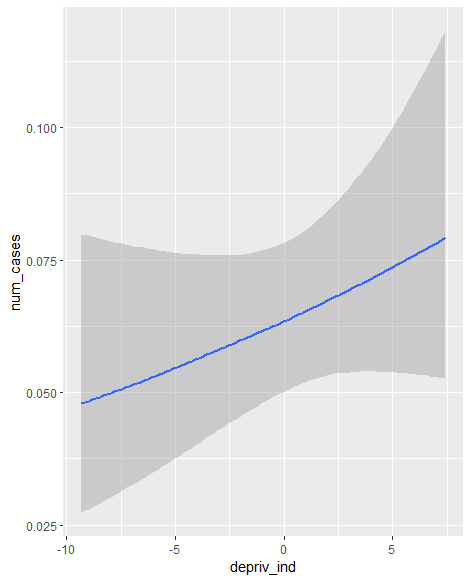 |
| Incidence | **Deprivation education** | **ML** | **0.13** | **0.06** | **0.00** | **0.26** | **1.00** | **10963** | **14651** | **100% (+)** | 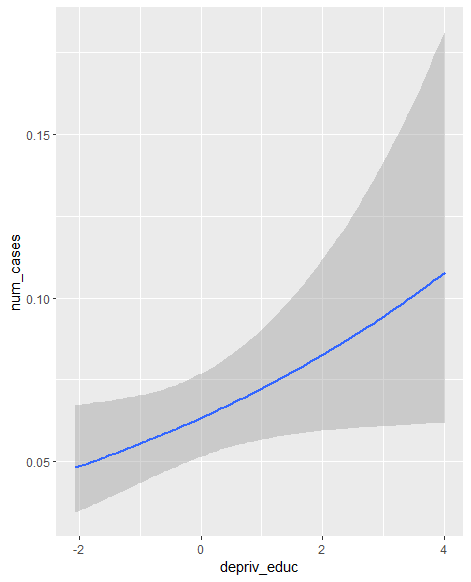 |
| Incidence | Economic inequality | ML | 0.57 | 1.31 | -2.04 | 3.08 | 1.00 | 19533 | 16781 | 67% (+) |  |
| Incidence | Migrant density (% migrants) | ML | -0.01 | 0.02 | -0.04 | 0.03 | 1.00 | 17915 | 16556 | 36% (+) |  |
| Incidence | **Area cannabis use** | **ML** | **0.15** | **0.08** | **-0.01** | **0.32** | **1.00** | **8049** | **10109** | **96% (+)** | 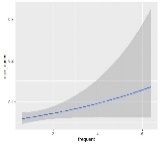 |

The table reports the association between each exposure and outcome. In particular, the column “*ML vs BYM2*” indicates whether the best fitting model was a Bayesian generalized hierarchical model (ML) or a Besag York Mollié spatial autocorrelation model (BYM2). Each row reports the median parameter value of the exposure, its 95% Credibility Interval (95% CI), Rhat value, effective sample sizes (ESS). The “*Probability coefficient non-zero*” column reports the posterior probability value for the hypothesis that the coefficient is above (+) or below (-) zero for the fixed effect. Finally, the “*Conditional effect”*  column reports the plot of the conditional effects of the exposures with 95% credibility bands. For instance, population density was estimated by a ML model and had a 99% posterior probability of being above zero.

### ST 3. Associations between exposures and outcomes: age at onset

| Outcome | Exposure | Estimate | Est.Error | l-95% CI | u-95% CI | Rhat | Bulk_ ESS | Tail_ ESS | Probability coefficient non-zero | Conditional effect |
| --- | --- | --- | --- | --- | --- | --- | --- | --- | --- | --- |
| Age at onset | Population density | -0.00 | 0.01 | -0.02 | 0.01 | 1.00 | 20373 | 16105 | 57% (-) |  |
| Age at onset | Deprivation | 0.00 | 0.00 | -0.00 | 0.01 | 1.00 | 17827 | 16249 | 79% (+) | 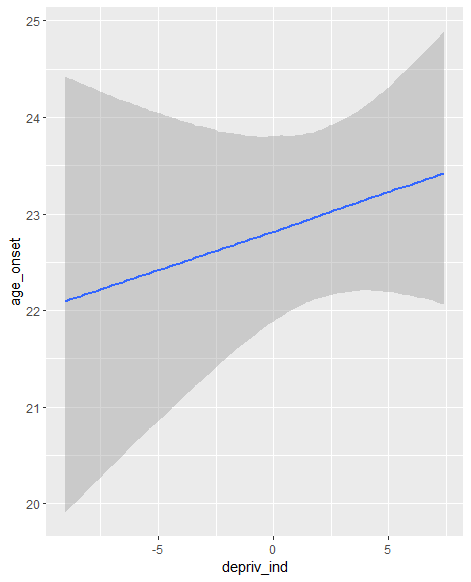 |
| Age at onset | Deprivation education | -0.00 | 0.01 | -0.02 | 0.02 | 1.00 | 12821 | 15293 | 46% (-) |  |
| Age at onset | Economic inequality | -0.13 | 0.25 | -0.63 | 0.35 | 1.00 | 12742 | 14541 | 70% (-) |  |
| Age at onset | Migrant density (% migrants) | 0.00 | 0.00 | -0.00 | 0.01 | 1.00 | 24538 | 17795 | 35% (-) |  |
| Age at onset | Area cannabis use | -0.02 | 0.02 | -0.05 | 0.02 | 1.00 | 11599 | 13546 | 82% (-) | 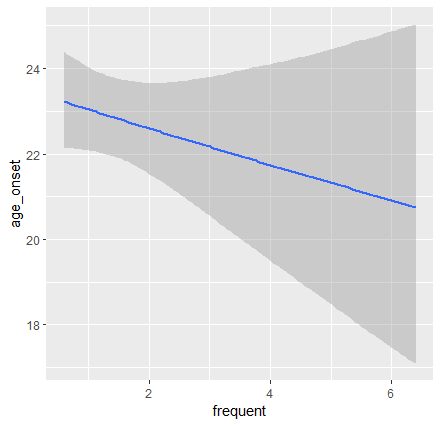 |

### ST 4. Associations between exposures and outcomes: migrant status

| Outcome | Exposure | Estimate | Est.Error | l-95% CI | u-95% CI | Rhat | Bulk_ ESS | Tail_ ESS | Probability coefficient non-zero | Conditional effect |
| --- | --- | --- | --- | --- | --- | --- | --- | --- | --- | --- |
| Migrant | Population density | 0.09 | 0.11 | -0.12 | 0.31 | 1.00 | 15039 | 14263 | 81% (+) | **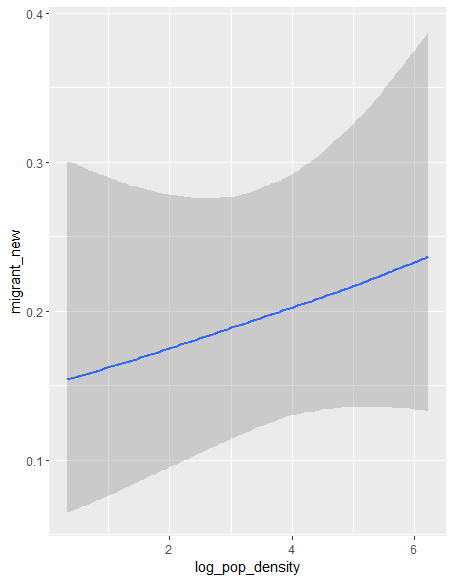** |
| Migrant | Deprivation | -0.03 | 0.06 | -0.14 | 0.08 | 1.00 | 16109 | 14135 | 69% (-) |  |
| Migrant | Deprivation education | 0.13 | 0.16 | -0.19 | 0.45 | 1.00 | 16202 | 13336 | 78% (+) |  |
| Migrant | Economic inequality | 5.03 | 3.12 | -1.23 | 11.10 | 1.00 | 10424 | 12780 | 95% (+) | 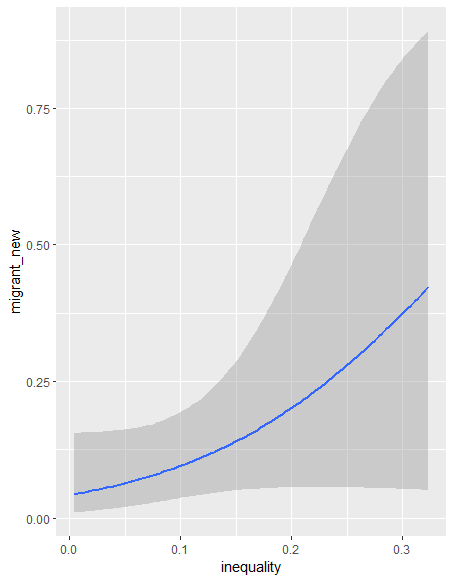 |
| Migrant | Migrant density (% migrants) | 0.10 | 0.04 | 0.03 | 0.18 | 1.00 | 11443 | 13452 | 100% | 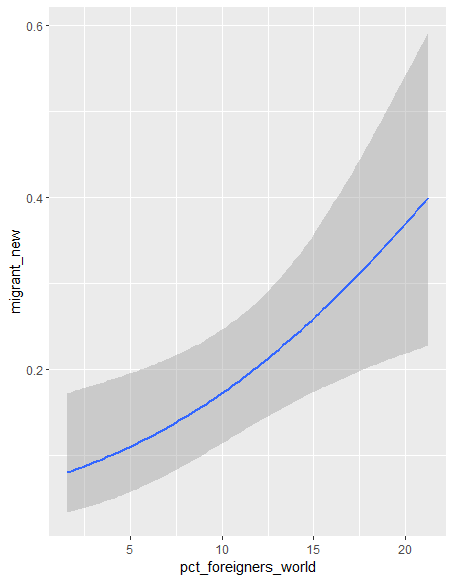 |
| Migrant | Area cannabis use | 0.20 | 0.23 | -0.27 | 0.65 | 1.00 | 11144 | 12157 | 82% | 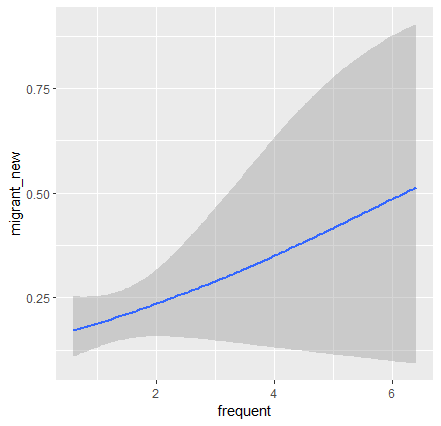 |

### ST 5 Associations between exposures and outcomes: unemployment

| Outcome | Exposure | Estimate | Est.Error | l-95% CI | u-95% CI | Rhat | Bulk_ ESS | Tail_ ESS | Probability coefficient non-zero | Conditional effect |
| --- | --- | --- | --- | --- | --- | --- | --- | --- | --- | --- |
| Unemployment | Population density | 0.06 | 0.07 | -0.08 | 0.21 | 1.00 | 14622 | 13504 | **80%** | 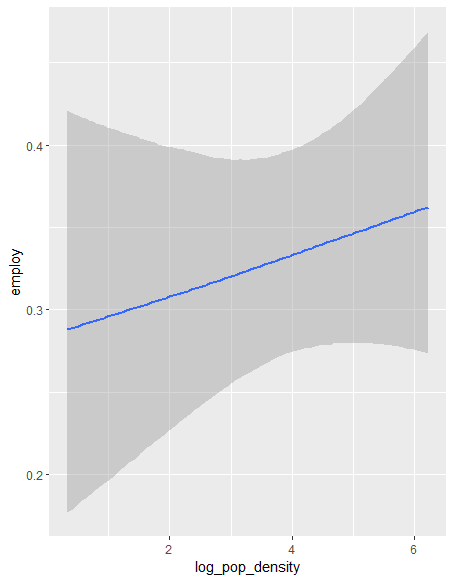 |
| Unemployment | Deprivation | 0.05 | 0.04 | -0.04 | 0.14 | 1.00 | 20987 | 16039 | 88% | 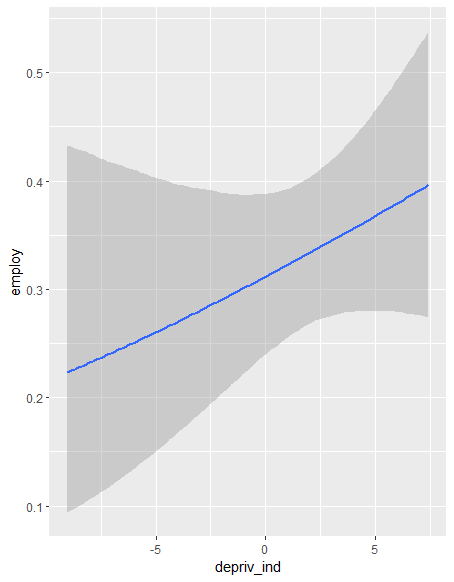 |
| Unemployment | Deprivation education | -0.05 | 0.12 | -0.29 | 0.18 | 1.00 | 10328 | 13440 | 68% (-) |  |
| Unemployment | Economic inequality | -1.08 | 2.39 | -5.89 | 3.54 | 1.00 | 11755 | 13060 | 68% (-) |  |
| Unemployment | Migrant density (% migrants) | -0.01 | 0.03 | -0.06 | 0.05 | 1.00 | 23578 | 15734 | 40% |  |
| Unemployment | Area cannabis use | 0.09 | 0.15 | -0.20 | 0.39 | 1.00 | 21214 | 14792 | 73% |  |

### ST 6. Associations between exposures and outcomes: substance use

| Outcome | Exposure | Estimate | Est.Error | l-95% CI | u-95% CI | Rhat | Bulk_ ESS | Tail_ ESS | Probability coefficient non-zero | Conditional effect |
| --- | --- | --- | --- | --- | --- | --- | --- | --- | --- | --- |
| Substance use | Population density | -0.14 | 0.07 | -0.28 | 0.01 | 1.00 | 18275 | 14324 | 97% (-) | **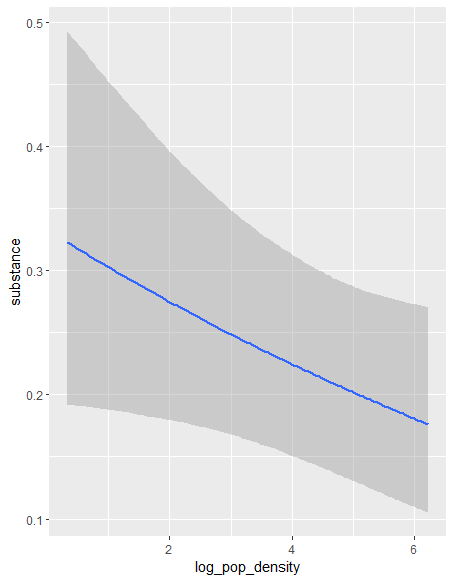** |
| Substance use | Deprivation | 0.09 | 0.04 | 0.01 | 0.18 | 1.00 | 23436 | 15885 | 98% (+) | 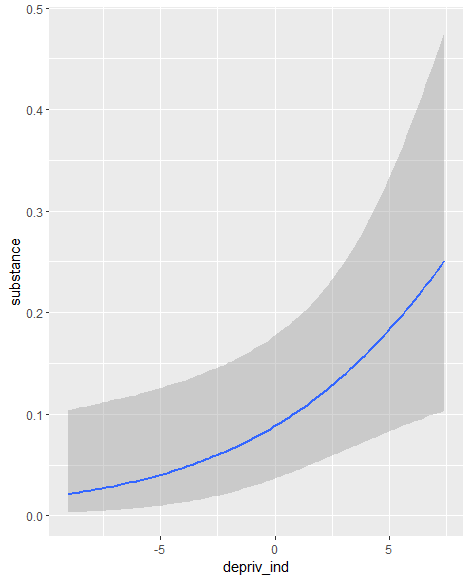 |
| Substance use | Deprivation education | 0.06 | 0.13 | -0.19 | 0.31 | 1.00 | 15802 | 15787 | 68% (+) |  |
| Substance use | Economic inequality | 0.79 | 2.56 | -4.22 | 5.79 | 1.00 | 13990 | 14736 | 62% (+) |  |
| Substance use | Migrant density (% migrants) | -0.03 | 0.03 | -0.08 | 0.03 | 1.00 | 15118 | 15683 | 81% (-) | 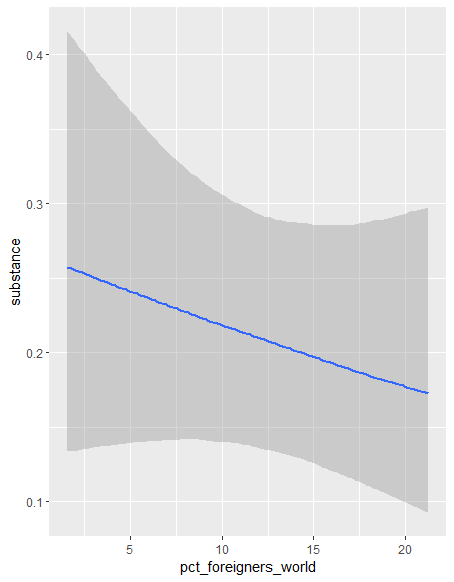 |
| Substance use | Area cannabis use | 0.20 | 0.21 | -0.25 | 0.59 | 1.00 | 9949 | 9559 | 85% (+) | 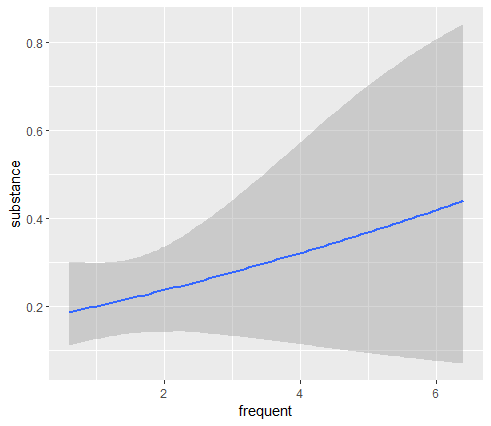 |

### ST 7. Associations between exposures and outcomes: DUP

| Outcome | Exposure | Estimate | Est.Error | l-95% CI | u-95% CI | Rhat | Bulk_ ESS | Tail_ ESS | Probability coefficient non-zero | Conditional effect |
| --- | --- | --- | --- | --- | --- | --- | --- | --- | --- | --- |
| DUP | Population density | 0.06 | -0.19 | 0.05 |  | 1.00 | 20385 | 15200 | 88% (-) | **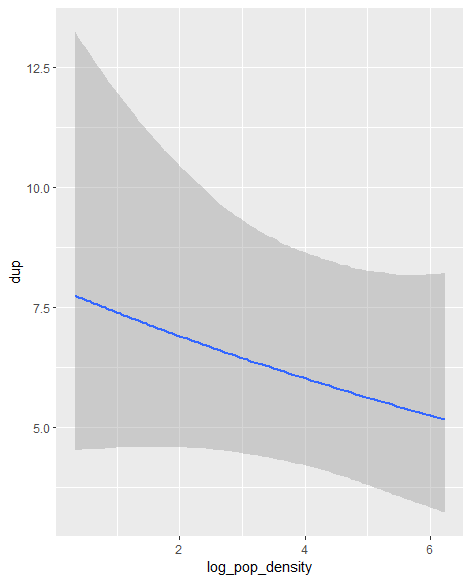** |
| DUP | Deprivation | -0.00 | 0.03 | -0.06 | 0.05 | 1.00 | 14457 | 15084 | 47% |  |
| DUP | Deprivation education | -0.02 | 0.08 | -0.18 | 0.15 | 1.00 | 11608 | 13473 | 41% |  |
| DUP | Economic inequality | 0.54 | 1.80 | -3.00 | 4.04 | 1.00 | 14426 | 14860 | 62% |  |
| DUP | Migrant density (% migrants) | -0.04 | 0.02 | -0.08 | 0.00 | 1.00 | 11681 | 14517 | 97% (-) | 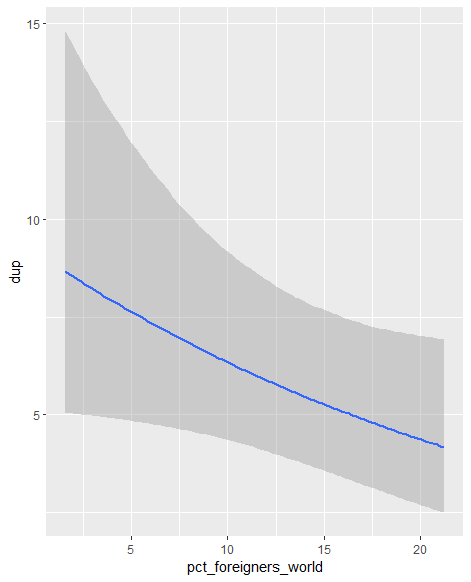 |
| DUP | Area cannabis use | 0.24 | 0.15 | -0.05 | 0.53 | 1.00 | 12825 | 13019 | 95% | 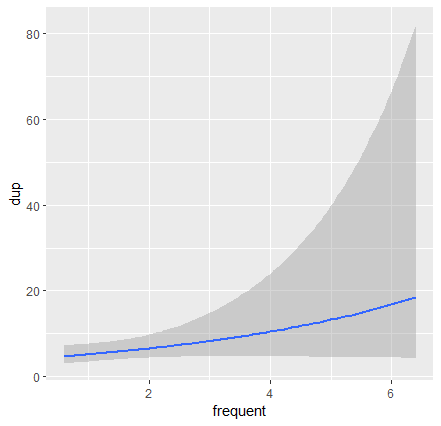 |

### ST 8. Associations between exposures and outcomes: Relational problems (HoNOS item 9)

| Outcome | Exposure | Estimate | Est.Error | l-95% CI | u-95% CI | Rhat | Bulk_ ESS | Tail_ ESS | Probability coefficient non-zero | Conditional effect |
| --- | --- | --- | --- | --- | --- | --- | --- | --- | --- | --- |
| Relational problems | Population density | -0.11 | 0.30 | -0.77 | 0.46 | 1.00 | 14450 | 9698 | **66%** |  |
| Relational problems | Deprivation | -0.20 | 0.22 | -0.70 | 0.17 | 1.00 | 9392 | 6781 | 85% (-) | 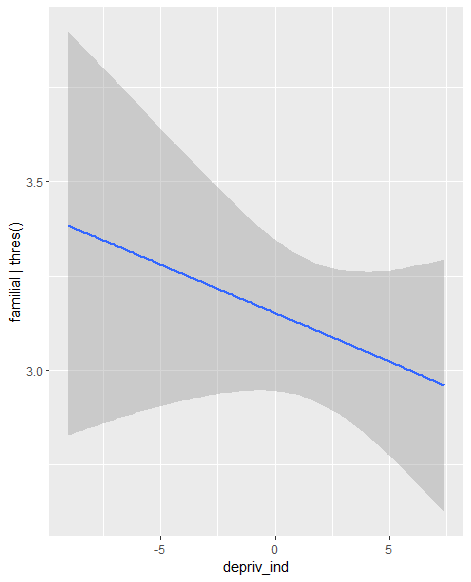 |
| Relational problems | Deprivation education | -0.45 | 0.76 | -2.13 | 0.92 | 1.00 | 6802 | 5735 | 74% (-) |  |
| Relational problems | Economic inequality | 11.19 | 12.74 | -10.74 | 41.09 | 1.00 | 6319 | 5880 | 84% (+) | 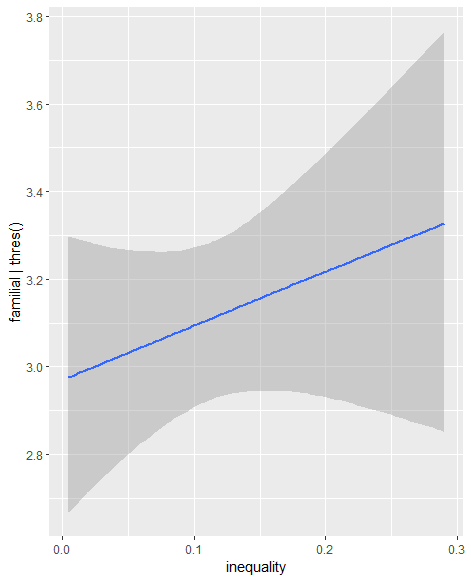 |
| Relational problems | Migrant density (% migrants) | -0.03 | 0.15 | -0.34 | 0.25 | 1.00 | 18623 | 11048 | 60% (-) |  |
| Relational problems | Area cannabis use | -0.08 | 1.39 | -2.88 | 2.84 | 1.00 | 7340 | 6207 | 47% (+) |  |

### ST 9. Associations between exposures and outcomes: resource problems (HoNOS item 11 and 12)

| Outcome | Exposure | Estimate | Est.Error | l-95% CI | u-95% CI | Rhat | Bulk_ ESS | Tail_ ESS | Probability coefficient non-zero | Conditional effect |
| --- | --- | --- | --- | --- | --- | --- | --- | --- | --- | --- |
| Resource problems | Population density | -0.30 | 0.27 | -0.91 | 0.16 | 1.00 | 5290 | 6041 | 90% (-) | 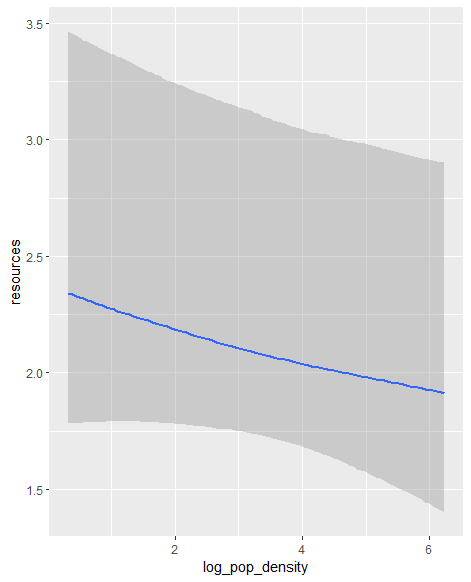 |
| Resource problems | Deprivation | -0.06 | 0.16 | -0.42 | 0.24 | 1.00 | 8570 | 8138 | 66% |  |
| Resource problems | Deprivation education | 0.38 | 0.96 | -1.43 | 2.48 | 1.00 | 6640 | 7167 | 67% (+) |  |
| Resource problems | Economic inequality | -7.33 | 17.42 | -46.53 | 24.42 | 1.00 | 8708 | 7790 | 68% |  |
| Resource problems | Migrant density (% migrants) | 0.07 | 0.19 | -0.31 | 0.48 | 1.00 | 11730 | 9776 | 64% |  |
| Resource problems | Area cannabis use | 0.07 | 0.57 | -1.06 | 1.23 | 1.00 | 4977 | 6260 | 56% (+) |  |

### ST 10. Summary of the associations with 80% or higher posterior probability of direction

| Outcomes | Exposure | Estimate | Est Error | l-95% CI | u-95% CI | Rhat | Bulk_ ESS | Tail_ ESS | Probability effect is over/ below zero | Conditional effect | Marginal effect ^2^ |
| --- | --- | --- | --- | --- | --- | --- | --- | --- | --- | --- | --- |
| Incidence | Deprivation | 0.03 | 0.03 | -0.02 | 0.08 | 1.00 | 16027 | 15201 | 89% | 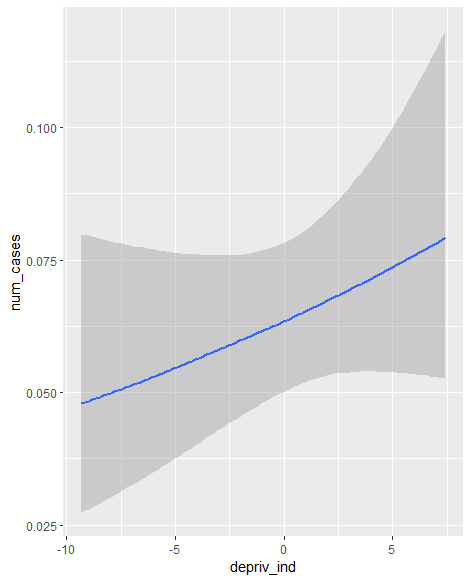 | Min – max: resp: 0.82 (-34.5; 12.4) ; IRR 3.47 (0.96; 39)  1sd: resp: 0.30 (-2.46; 2.17); IRR 1.13 (0.96; 1.34) |
| Age at onset | Area cannabis use | -0.02 | 0.02 | -0.05 | 0.02 | 1.00 | 11599 | 13546 | 82% (-) | 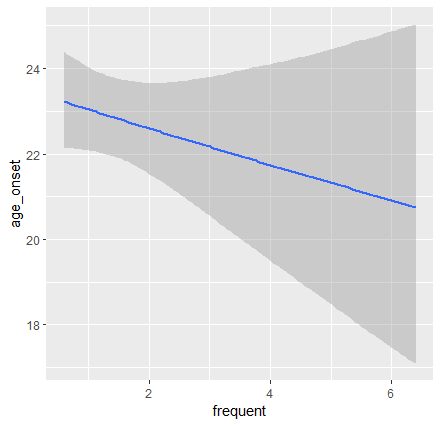 | Min – max: resp: -2.56 (-7.8; 2.23)  1sd: resp: -0.78 (-2.32; 0.69) |
| Unemployment | Population density | 0.06 | 0.07 | -0.08 | 0.21 | 1.00 | 14622 | 13504 | **80%** | 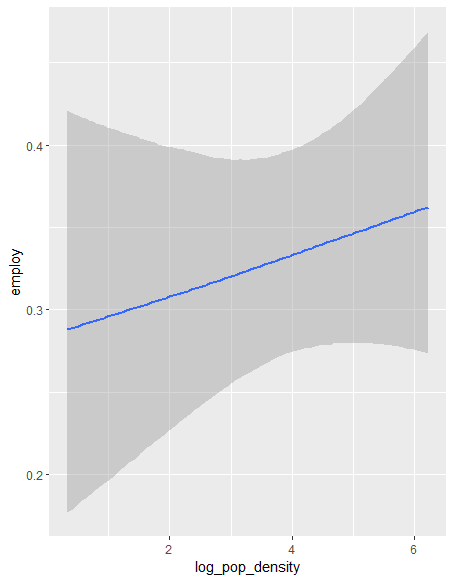 | Min – max: resp: 7% (-11%; 26%); OR 1.24 (0.77; 2.31)  1sd: resp: 1% (-2%; 5%); OR 1.04 (0.95; 1.15) |
| Unemployment | Deprivation | 0.05 | 0.04 | -0.04 | 0.14 | 1.00 | 20987 | 16039 | 88% | 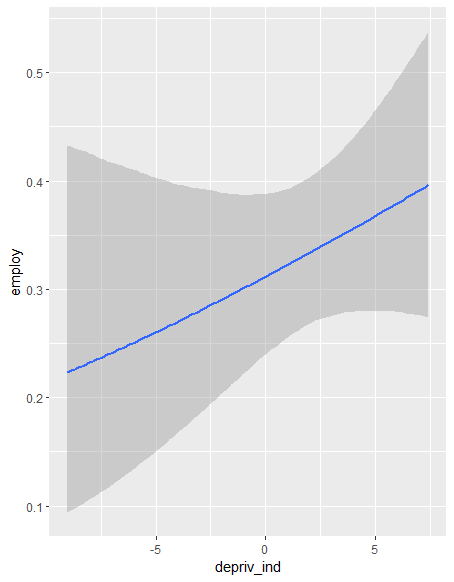 | Min – max: resp: 16% (-15%; 42%); OR 2.29 (0.85; 20.3)  1sd: resp: 2% (-2%; 7%); OR 1.07 (0.96; 1.20) |
| Migrant status | Population density | 0.09 | 0.11 | -0.12 | 0.31 | 1.00 | 15039 | 14263 | **81%** | **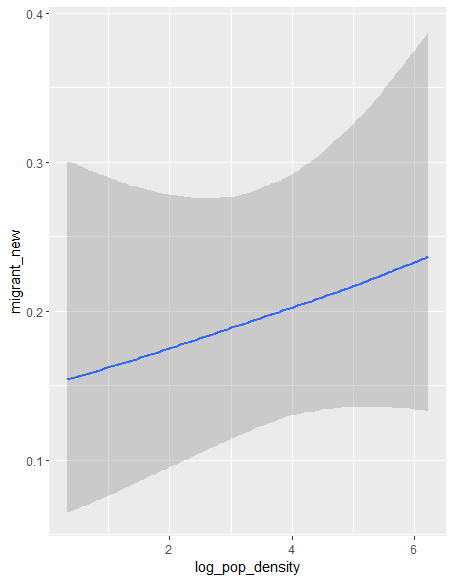** | Min – max: resp: 8% (-12%; 25%); OR 1.80 (0.68; 17.7)  1sd: resp: 2% (-2%; 6%); OR 1.09 (0.91; 1.35) |
| Migrant status | Area cannabis use | 0.20 | 0.23 | -0.27 | 0.65 | 1.00 | 11144 | 12157 | 82% | 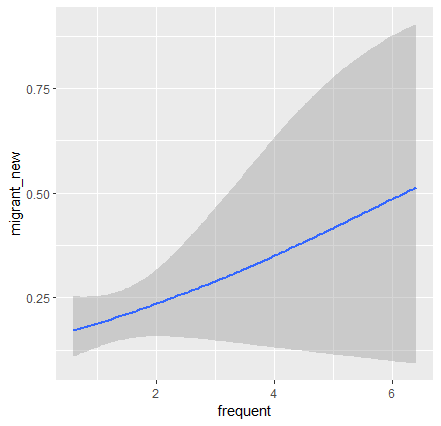 | Min – max: resp: 25% (-14%; 50%); OR 2.68 (0.36; 21.6)  1sd: resp: 8.6% (4.2%; 18%); OR 1.47 (0.81; 2.60) |
| Substance use | Area cannabis use | 0.20 | 0.21 | -0.25 | 0.59 | 1.00 | 9949 | 9559 | 85% | 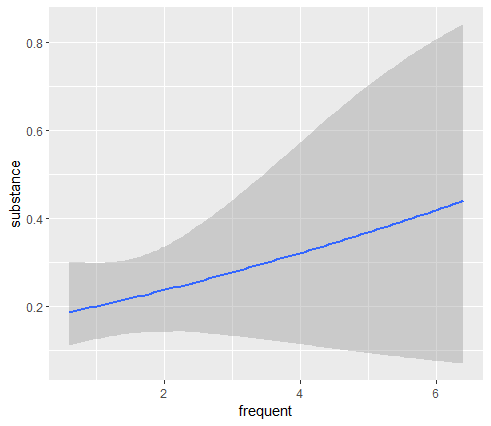 | Min – max: resp: 26% (-28%; 60%); OR 2.22 (0.49; 9.63)  1sd: resp: 8.3% (-8.9%; 22.3%); OR 1.25 (0.78; 1.85) |
| DUP | **Population density** | 0.06 | -0.19 | 0.05 |  | 1.00 | 20385 | 15200 | **88%** | **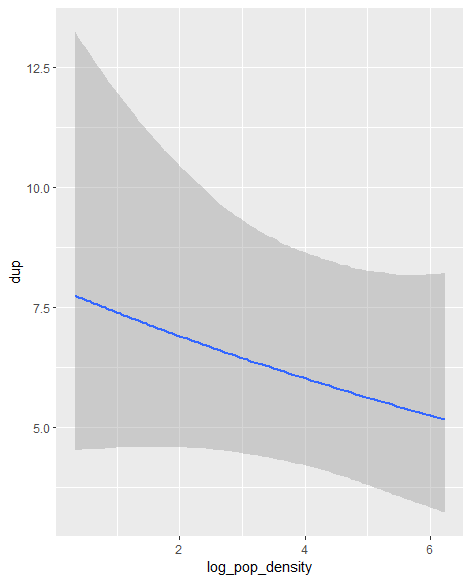** | Min – max: resp: -13.7 (-5651; 39.4)  1sd: resp: -2.18 months (-916; 8.63) |
| Relational problems | Deprivation | -0.20 | 0.22 | -0.70 | 0.17 | 1.00 | 9392 | 6781 | 85% (-) | 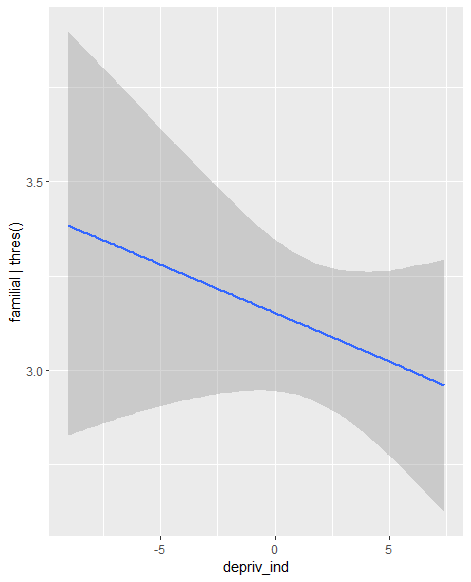 | Min – max: resp: for each level   1. 3.2% (-8.9%; 50%) 2. 6.9% (-7.4%; 19.8%) 3. 4.9% (-1.1%; 12.4%) 4. -4.7% (-15.7%; 8.7%) 5. -9.6% (-28.9%; 6.71%)   1sd: resp: for each level   1. 6.0% (-0.5%; 1.7%) 2. 1.1% (-1.2%; 3.3%) 3. 0.3% (-1.1%; 1.4%) 4. -1.1% (-3.3%; 1.1%) 5. -0.9% (-2.9%; 1.4%)   OR (1sd) for each level   1. 1.10 (0.93; 1.30) 2. 1.07 (0.94; 1.20) 3. 1.01 (0.97; 1.05) 4. 0.96 (0.89; 1.05) 5. 0.94 (0.81; 1.12) |
| Relational problems | Economic inequality | 11.19 | 12.74 | -10.74 | 41.09 | 1.00 | 6319 | 5880 | 84% (+) | 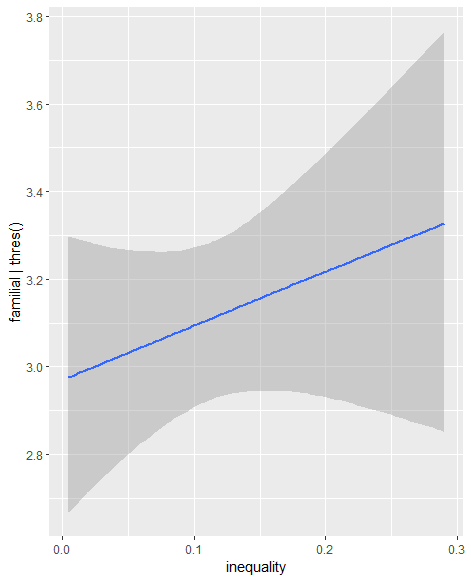 | Min – max: resp: for each level   1. -3.5% (-10.4%; 4.0%) 2. -7.1% (-19.0%; 7.0%) 3. -3.1% (-9.3%; 2.1%) 4. 6.9% (-7.1%; 16.7%) 5. 6.8% (-6.8%; 21.6%)   1sd: resp: for each level   1. -0.8% (-2.6%; 0.5%) 2. -1.7% (-4.8%; 1.6%) 3. -0.6% (-1.9%; 0.6%) 4. 1.7% (-1.6%; 4.7%) 5. 1.5% (-1.5%; 4.4%)   OR (1sd) for each level   1. 0.88 (0.69; 1.13) 2. 0.91 (0.77; 1.09) 3. 0.98 (0.93; 1.02) 4. 1.06 (0.94; 1.19) 5. 1.12 (0.89; 1.40) |
| Resource problems | **Population density** | **-0.30** | **0.27** | **-0.91** | **0.16** | **1.00** | **5290** | **6041** | **90% (-)** | 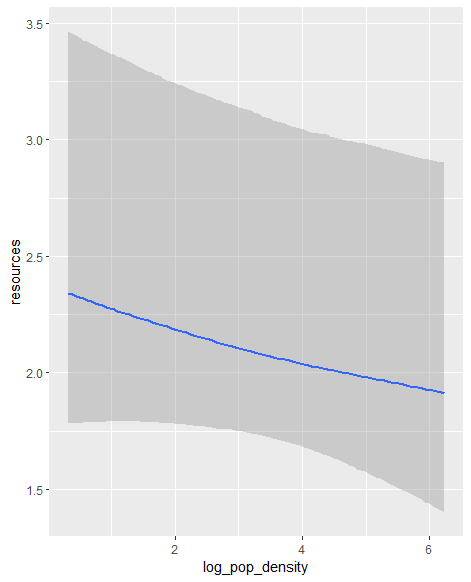 | Min – max: resp: for each level   1. 11.4% (-8.0%; 28.9%) 2. 1.3% (-1.0%; 7.7%) 3. -4.0% (-10.1%; 3.5%) 4. -4.8% (-12.4%; 2.2%) 5. -3.9% (-12.8%; 1.6%)   1sd: resp: for each level   1. 2.4% (-1.6%; 6.4%) 2. 0.04% (-0.7%; 1.4%) 3. -0.9% (-2.7%; 0.6%) 4. -0.9% (-2.4%; 0.5%) 5. -0.6% (-1.9%; 0.4%)     OR (1sd) for each level   1. 1.48 (0.93; 711) 2. 1.14 (0.98; 7818) 3. 0.95 (0.81; NA) 4. 0.86 (0.62; NA) 5. 0.82 (0.53; NA) |

We did not find consistent effects of socioeconomic indices on **age at onset,** except for a possible role of area-level cannabis use (ST 4). Whereas, among effects with lower posterior probability of being significant (ST 5), higher population density was associated with higher likelihood of being a **migrant** (81% probability; OR (1sd): 1.09; 95% CrI: 0.91; 1.35). Population density and global deprivation were associated with small increases of the likelihood of being **unemployed** (density: 80% probability; OR for an sd increase: 1.04; 95% CrI: 0.95; 1.15; deprivation: 88% probability; OR: 1.07; 95% CrI: 0.96; 1.20; ST 4 and ST11). The migrant density (% migrants) in the area predicted a reduced likelyhood of **substance use** among FEP (81% probability of negative effect). Higher population density was associated with a shorter **DUP** (difference for 1sd: -2.18 months; 95% CrI:-916; 8.63), whereas the prevalence of province-level frequent use of cannabis was associated with a longer DUP (Difference (1sd): 12.9 months; ; 95% CrI: -2.86; 6229). Global deprivation was associated with a tendency to lower ratings relative to Relational problems measured with the HoNOS, while economic inequality was associated with higher ratings (84% and 85% probability, respectively, ST 11). Finally, higher population density predicted a tendency to lower ratings in the area of resource problems (items 11 and 12 of the HoNOS, 84% probability).

# Supplementary References

Belvederi Murri, Martino, e Mario Amore. 2018. «The Multiple Dimensions of Insight in Schizophrenia-Spectrum Disorders». *Schizophrenia Bulletin*. doi: 10.1093/schbul/sby092.

Belvederi Murri, Martino, Luigi Grassi, Rosangela Caruso, Maria Giulia Nanni, Luigi Zerbinati, Sylke Andreas, Berta Ausín, Alessandra Canuto, Martin Härter, Manuel Muñoz Lopez, Kerstin Weber, Hans-Ulrich Wittchen, Jana Volkert, e George S. Alexopoulos. 2022. «Depressive Symptom Complexes of Community-Dwelling Older Adults: A Latent Network Model.» *Molecular Psychiatry* 27(2):1075–82. doi: 10.1038/s41380-021-01310-y.

Benedetti, Elisa, Giuliano Resce, Paolo Brunori, e Sabrina Molinaro. 2021. «Cannabis Policy Changes and Adolescent Cannabis Use: Evidence from Europe». *International Journal of Environmental Research and Public Health* 18(10):5174. doi: 10.3390/ijerph18105174.

van Bork, Riet, Mijke Rhemtulla, Lourens J. Waldorp, Joost Kruis, Shirin Rezvanifar, e Denny Borsboom. 2021. «Latent Variable Models and Networks: Statistical Equivalence and Testability». *Multivariate Behavioral Research* 56(2):175–98. doi: 10.1080/00273171.2019.1672515.

Boydell, J., J. van Os, K. McKenzie, J. Allardyce, R. Goel, R. G. McCreadie, e R. M. Murray. 2001. «Incidence of Schizophrenia in Ethnic Minorities in London: Ecological Study into Interactions with Environment». *BMJ (Clinical Research Ed.)* 323(7325):1336–38. doi: 10.1136/bmj.323.7325.1336.

Bürkner, Paul-Christian, Jonah Gabry, e Aki Vehtari. 2021. «Efficient leave-one-out cross-validation for Bayesian non-factorized normal and Student-t models». *Computational Statistics* 36(2):1243–61. doi: 10.1007/s00180-020-01045-4.

Caranci, Nicola, Annibale Biggeri, Laura Grisotto, Barbara Pacelli, Teresa Spadea, e Giuseppe Costa. 2010. «[The Italian deprivation index at census block level: definition, description and association with general mortality]». *Epidemiologia E Prevenzione* 34(4):167–76.

Comacchio, Carla, Louise M. Howard, Chiara Bonetto, Riccardo Lo Parrino, Karin Furlato, Enrico Semrov, Antonio Preti, Luca Mesiano, Giovanni Neri, Giovanni De Girolamo, Katia de Santi, Elisabetta Miglietta, Sarah Tosato, Doriana Cristofalo, Antonio Lasalvia, Mirella Ruggeri, e GET UP Group. 2019. «The Impact of Gender and Childhood Abuse on Age of Psychosis Onset, Psychopathology and Needs for Care in Psychosis Patients». *Schizophrenia Research* 210:164–71. doi: 10.1016/j.schres.2018.12.046.

Comacchio, Carla, Antonio Lasalvia, e Mirella Ruggeri. 2019. «Current Evidence of Childhood Traumatic Experiences in Psychosis - Focus on Gender Differences». *Psychiatry Research* 281:112507. doi: 10.1016/j.psychres.2019.112507.

Di Forti, Marta, Diego Quattrone, Tom P. Freeman, Giada Tripoli, Charlotte Gayer-Anderson, Harriet Quigley, Victoria Rodriguez, Hannah E. Jongsma, Laura Ferraro, Caterina La Cascia, Daniele La Barbera, Ilaria Tarricone, Domenico Berardi, Andrei Szöke, Celso Arango, Andrea Tortelli, Eva Velthorst, Miguel Bernardo, Cristina Marta Del-Ben, Paulo Rossi Menezes, Jean-Paul Selten, Peter B. Jones, James B. Kirkbride, Bart Pf Rutten, Lieuwe de Haan, Pak C. Sham, Jim van Os, Cathryn M. Lewis, Michael Lynskey, Craig Morgan, Robin M. Murray, e EU-GEI WP2 Group. 2019. «The Contribution of Cannabis Use to Variation in the Incidence of Psychotic Disorder across Europe (EU-GEI): A Multicentre Case-Control Study». *The Lancet. Psychiatry* 6(5):427–36. doi: 10.1016/S2215-0366(19)30048-3.

Donegan, Connor. 2022. «Geostan: An R Package for Bayesian Spatial Analysis». *Journal of Open Source Software* 7(79):4716. doi: 10.21105/joss.04716.

Eliasson, Emma T., Lily McNamee, Linda Swanson, Stephen M. Lawrie, e Matthias Schwannauer. 2021. «Unpacking Stigma: Meta-Analyses of Correlates and Moderators of Personal Stigma in Psychosis». *Clinical Psychology Review* 89:102077. doi: 10.1016/j.cpr.2021.102077.

Ferrara, Maria, Enrico Tedeschini, Flavia Baccari, Vincenzo Musella, Francesca Vacca, Fausto Mazzi, Mila Ferri, Vinod Srihari, Fabrizio Starace, e Early Psychosis Working Group. 2019. «Early Intervention Service for First Episode Psychosis in Modena, Northern Italy: The First Hundred Cases». *Early Intervention in Psychiatry* 13(4):1011–17. doi: 10.1111/eip.12788.

Golino, Hudson F., e Alexander P. Christensen. 2020. «Random, Factor, or Network Model? Predictions From Neural Networks». *PsyArXiv*. doi: 10.31234/osf.io/awkcb.

Grover, Sandeep, Natarajan Varadharajan, e Sandesh Venu. 2024. «Urbanization and Psychosis: An Update of Recent Evidence». *Current Opinion in Psychiatry* 37(3):191–201. doi: 10.1097/YCO.0000000000000931.

ISTAT Italian Institute of Statistics. 2020. «Resident population data». *Popolazione residente comunale per sesso, anno di nascita e stato civile*.

Jongsma, Hannah E., Charlotte Gayer-Anderson, Antonio Lasalvia, Diego Quattrone, Alice Mulè, Andrei Szöke, Jean Paul Selten, Caitlin Turner, Celso Arango, Ilaria Tarricone, Domenico Berardi, Andrea Tortelli, Pierre Michel Llorca, Lieuwe De Haan, Julio Bobes, Miguel Bernardo, Julio Sanjuán, José Luis Santos, Manuel Arrojo, Cristina Marta Del-Ben, Paulo Rossi Menezes, Robin M. Murray, Bart P. Rutten, Peter B. Jones, Jim Van Os, Craig Morgan, James B. Kirkbride, Ulrich Reininghaus, Marta Di Forti, Kathryn Hubbard, Stephanie Beards, Simona A. Stilo, Giada Tripoli, Mara Parellada, Pedro Cuadrado, José Juan Rodríguez Solano, Angel Carracedo, Enrique García Bernardo, Laura Roldán, Gonzalo López, Bibiana Cabrera, Esther Lorente-Rovira, Paz Garcia-Portilla, Javier Costas, Estela Jiménez-López, Mario Matteis, Marta Rapado, Emiliano González, Covadonga Martínez, Emilio Sánchez, Ma Soledad Olmeda, Nathalie Franke, Eva Velthorst, Fabian Termorshuizen, Daniella Van Dam, Elsje Van Der Ven, Elles Messchaart, Marion Leboyer, Franck Schürhoff, Stéphane Jamain, Flora Frijda, Grégoire Baudin, Aziz Ferchiou, Baptiste Pignon, Jean Romain Richard, Thomas Charpeaud, Anne Marie Tronche, Daniele La Barbera, Caterina La Cascia, Giovanna Marrazzo, Lucia Sideli, Crocettarachele Sartorio, Laura Ferraro, Fabio Seminerio, Camila Marcelino Loureiro, Rosana Shuhama, Mirella Ruggeri, Sarah Tosato, Chiara Bonetto, e Doriana Cristofalo. 2018. «Treated incidence of psychotic disorders in the multinational EU-GEI study». *JAMA Psychiatry* 75(1):36–46. doi: 10.1001/jamapsychiatry.2017.3554.

Jongsma, Hannah E., Caitlin Turner, James B. Kirkbride, e Peter B. Jones. 2019. «International incidence of psychotic disorders, 2002–17: a systematic review and meta-analysis». *The Lancet Public Health* 4(5):e229–44. doi: 10.1016/S2468-2667(19)30056-8.

Kirkbride, James B., Deidre M. Anglin, Ian Colman, Jennifer Dykxhoorn, Peter B. Jones, Praveetha Patalay, Alexandra Pitman, Emma Soneson, Thomas Steare, Talen Wright, e Siân Lowri Griffiths. 2024. «The Social Determinants of Mental Health and Disorder: Evidence, Prevention and Recommendations». *World Psychiatry: Official Journal of the World Psychiatric Association (WPA)* 23(1):58–90. doi: 10.1002/wps.21160.

Kudva, Kundadak Ganesh, Samer El Hayek, Anoop Krishna Gupta, Shunya Kurokawa, Liu Bangshan, Maria Victoria C. Armas-Villavicencio, Kengo Oishi, Saumya Mishra, Saratcha Tiensuntisook, e Norman Sartorius. 2020. «Stigma in Mental Illness: Perspective from Eight Asian Nations». *Asia-Pacific Psychiatry: Official Journal of the Pacific Rim College of Psychiatrists* 12(2):e12380. doi: 10.1111/appy.12380.

Large, Matthew, Saeed Farooq, Olav Nielssen, e Tim Slade. 2008. «Relationship between Gross Domestic Product and Duration of Untreated Psychosis in Low- and Middle-Income Countries». *The British Journal of Psychiatry: The Journal of Mental Science* 193(4):272–78. doi: 10.1192/bjp.bp.107.041863.

Lipsky, Ari M., e Sander Greenland. 2022. «Causal Directed Acyclic Graphs». *JAMA* 327(11):1083–84. doi: 10.1001/jama.2022.1816.

Logeswaran, Yanakan, Jennifer Dykxhoorn, Christina Dalman, e James B. Kirkbride. 2023. «Social Deprivation and Population Density Trajectories Before and After Psychotic Disorder Diagnosis». *JAMA Psychiatry* 80(12):1258–68. doi: 10.1001/jamapsychiatry.2023.3220.

Lora, A., G. Bai, S. Bianchi, G. Bolongaro, G. Civenti, A. Erlicher, G. Maresca, E. Monzani, B. Panetta, D. Von Morgen, F. Rossi, V. Torri, e P. Morosini. 2001. «The italian version of HoNOS (Health of the Nation Outcome Scales), a scale for evaluating the outcome and the severity in mental health services». *Epidemiologia e Psichiatria Sociale*. doi: 10.1017/s1121189x00005339.

Marbin, Derin, Stefan Gutwinski, Stefanie Schreiter, e Andreas Heinz. 2022. «Perspectives in Poverty and Mental Health». *Frontiers in Public Health* 10. doi: 10.3389/fpubh.2022.975482.

March, Dana, Stephani L. Hatch, Craig Morgan, James B. Kirkbride, Michaeline Bresnahan, Paul Fearon, e Ezra Susser. 2008. «Psychosis and Place». *Epidemiologic Reviews* 30:84–100. doi: 10.1093/epirev/mxn006.

Morgan, Nirvana, e Sumaya Mall. 2019. «Pathways between Urbanization and Harmful Substance Use». *Current Opinion in Psychiatry* 32(3):218–23. doi: 10.1097/YCO.0000000000000488.

Morris, Mitzi, Katherine Wheeler-Martin, Dan Simpson, Stephen J. Mooney, Andrew Gelman, e Charles DiMaggio. 2019. «Bayesian Hierarchical Spatial Models: Implementing the Besag York Mollié Model in Stan». *Spatial and Spatio-Temporal Epidemiology* 31:100301. doi: 10.1016/j.sste.2019.100301.

O’Donoghue, Brian, Eric Roche, e Abbie Lane. 2016. «Neighbourhood Level Social Deprivation and the Risk of Psychotic Disorders: A Systematic Review». *Social Psychiatry and Psychiatric Epidemiology* 51(7):941–50. doi: 10.1007/s00127-016-1233-4.

OECD Data. 2024. «Income Inequality - OECD Data». *OECD Data*. Recuperato 8 aprile 2024 (http://data.oecd.org/inequality/income-inequality.htm).

Pearce, Eiluned, Mary Birken, Sarah Pais, Millie Tamworth, Yutung Ng, Jingyi Wang, Beverley Chipp, Ellena Crane, Merle Schlief, Jinyan Yang, Aggelos Stamos, Lui Kwan Cheng, Maria Condon, Brynmor Lloyd-Evans, James B. Kirkbride, David Osborn, Alexandra Pitman, e Sonia Johnson. 2023. «Associations between Constructs Related to Social Relationships and Mental Health Conditions and Symptoms: An Umbrella Review». *BMC Psychiatry* 23(1):652. doi: 10.1186/s12888-023-05069-0.

Pickett, Kate E., e Richard G. Wilkinson. 2010. «Inequality: An Underacknowledged Source of Mental Illness and Distress». *The British Journal of Psychiatry: The Journal of Mental Science* 197(6):426–28. doi: 10.1192/bjp.bp.109.072066.

Piketty, Thomas, e Emmanuel Saez. 2014. «Inequality in the long run». *Science* 344(6186):838–43. doi: 10.1126/science.1251936.

Reichert, A., e R. Jacobs. 2018. «Socioeconomic Inequalities in Duration of Untreated Psychosis: Evidence from Administrative Data in England». *Psychological Medicine* 48(5):822–33. doi: 10.1017/S0033291717002197.

Reiss, Franziska. 2013. «Socioeconomic inequalities and mental health problems in children and adolescents: A systematic review». *Social Science & Medicine* 90:24–31. doi: 10.1016/j.socscimed.2013.04.026.

Riebler, Andrea, Sigrunn H. Sørbye, Daniel Simpson, e Håvard Rue. 2016. «An intuitive Bayesian spatial model for disease mapping that accounts for scaling».

Rosano, Aldo, Barbara Pacelli, Nicolás Zengarini, Giuseppe Costa, Cesare Cislaghi, e Nicola Caranci. 2020. «[Update and review of the 2011 Italian deprivation index calculated at the census section level]». *Epidemiologia E Prevenzione* 44(2–3):162–70. doi: 10.19191/EP20.2-3.P162.039.

Rosoff, Daniel B., Zachary A. Kaminsky, Andrew M. McIntosh, George Davey Smith, e Falk W. Lohoff. 2020. «Educational Attainment Reduces the Risk of Suicide Attempt among Individuals with and without Psychiatric Disorders Independent of Cognition: A Bidirectional and Multivariable Mendelian Randomization Study with More than 815,000 Participants». *Translational Psychiatry* 10(1):1–15. doi: 10.1038/s41398-020-01047-2.

Samo, Andrew, Alexander P. Christensen, Francisco José Abad, Luis Eduardo Garrido, Marcos Jiménez, Eduardo Garcia-Garzon, Hudson Golino, e Samuel T. McAbee. 2022. «Hierarchical EGA of Personality».

Sarraf, Lisa, Martin Lepage, e Geneviève Sauvé. 2022. «The Clinical and Psychosocial Correlates of Self-Stigma among People with Schizophrenia Spectrum Disorders across Cultures: A Systematic Review and Meta-Analysis». *Schizophrenia Research* 248:64–78. doi: 10.1016/j.schres.2022.08.001.

Schultze-Lutter, F., C. Michel, S. J. Schmidt, B. G. Schimmelmann, N. P. Maric, R. K. R. Salokangas, A. Riecher-Rössler, M. van der Gaag, M. Nordentoft, A. Raballo, A. Meneghelli, M. Marshall, A. Morrison, S. Ruhrmann, e J. Klosterkötter. 2015. «EPA guidance on the early detection of clinical high risk states of psychoses». *European Psychiatry*. doi: 10.1016/j.eurpsy.2015.01.010.

Serafini, Alessio, e Giancarlo Ferrara. 2023. *mapping: Automatic Download, Linking, Manipulating Coordinates for Maps*.

Stilo, Simona A., e Robin M. Murray. 2019. «Non-Genetic Factors in Schizophrenia». *Current Psychiatry Reports* 21(10):100. doi: 10.1007/s11920-019-1091-3.

Sum, Min Yi, Charmaine Tsz Wing Wong, Sin Ting Chu, Angel Li, Athena Hoi Ting Lee, Eric Yu Hai Chen, e Sherry Kit Wa Chan. 2024. «Systematic Review and Meta-Analysis of Internalised Stigma and Stigma Resistance in Patients with Psychosis: The Impact of Individualism-Collectivism Culture and Other Individual Factors». *The International Journal of Social Psychiatry* 207640231216924. doi: 10.1177/00207640231216924.

Textor, Johannes, Juliane Hardt, e Sven Knüppel. 2011. «DAGitty: A Graphical Tool for Analyzing Causal Diagrams». *Epidemiology* 22(5):745. doi: 10.1097/EDE.0b013e318225c2be.

Tibber, Marc S., Fahreen Walji, James B. Kirkbride, e Vyv Huddy. 2022. «The Association between Income Inequality and Adult Mental Health at the Subnational Level—a Systematic Review». *Social Psychiatry and Psychiatric Epidemiology* 57(1):1–24. doi: 10.1007/s00127-021-02159-w.

Westreich, Daniel, e Sander Greenland. 2013. «The Table 2 Fallacy: Presenting and Interpreting Confounder and Modifier Coefficients». *American Journal of Epidemiology* 177(4):292–98. doi: 10.1093/aje/kws412.
